# Kidney function and brain health: cognitive and structural signatures across cohorts

**DOI:** 10.1002/alz.71840

**Published:** 2026-09-23

**Authors:** Min Li, Wenbo Yang, Ying Hui, Huijing Shi, Yuntao Wu, Li Yu, Lijun Song, Yanfang Tan, Xintian Ren, Xiaoyan Bai, Xiaoshuai Li, Zhenghan Yang, Weikang Guo, Shouling Wu, Jing Li, Zhenchang Wang, Han Lv, Hao Wang

**Affiliations:** ^1^ Clinical Epidemiology and EBM Unit Beijing Friendship Hospital Capital Medical University Beijing China; ^2^ Department of Radiology Beijing Friendship Hospital Capital Medical University Beijing China; ^3^ Department of Radiology Kailuan General Hospital Tangshan Hebei China; ^4^ Department of Nephrology Beijing Friendship Hospital Capital Medical University Beijing China; ^5^ Department of Cardiology Kailuan General Hospital Tangshan Hebei China; ^6^ Department of Radiology School of Clinical Medicine Beijing Tsinghua Changgung Hospital Tsinghua University Beijing China; ^7^ Precision and Intelligence Medical Imaging Lab Beijing Friendship Hospital Capital Medical University Beijing China; ^8^ Beijing Key Laboratory of Innovation and Transformation of Specialized CT/MR Imaging Equipment Beijing China

**Keywords:** brain structure, cognitive function, cumulative, estimated glomerular filtration rate, variability

## Abstract

**INTRODUCTION:**

Chronic kidney disease (CKD) is linked to cognitive decline, yet the roles of estimated glomerular filtration rate (eGFR) and its variability remain unclear.

**METHODS:**

We analyzed 6847 adults from two population‐based cohorts (the Multimodality MEdical imaging sTudy bAsed on KaiLuan Study [META‐KLS], UK Biobank) and 426 CKD patients from Beijing Friendship Hospital (BFH)‐CKD. Exposures included eGFR level and variability. Cognitive function was assessed using the Montreal Cognitive Assessment (MoCA) and brain structure by magnetic resonance imaging (MRI).

**RESULTS:**

In META‐KLS, higher cumulative eGFR was associated with higher MoCA scores (*β* = 0.350; 95% confidence interval [CI]: 0.148 to 0.552), whereas greater eGFR variability was associated with lower MoCA scores (*β* = −0.454; 95% CI: −0.659 to −0.250). Across cohorts, higher eGFR was associated with white matter, superior frontal, and middle temporal volumes. In META‐KLS, greater variability showed nominal inverse associations with inferior parietal and middle temporal volumes and reduced rostral anterior cingulate cortex thickness.

**DISCUSSION:**

Lower eGFR and greater eGFR variability were associated with lower MoCA scores and region‐specific structural brain alterations.

## Highlights

- Lower eGFR and greater eGFR variability were associated with poorer cognition.
- Lower eGFR was linked to smaller WM, middle temporal, and superior frontal volumes.
- Greater eGFR variability was linked to regional cortical structural changes.
- Middle temporal volume partly explained eGFR–cognition associations.

## 1 BACKGROUND

Dementia is a major and rapidly growing public health challenge. In 2018, an estimated 50 million people worldwide were living with dementia, and the number is expected to rise to 152 million by 2050.^1^, ^2^ Chronic kidney disease (CKD) has emerged as an independent risk factor for cognitive impairment.^3^ Patients with CKD not only have an increased risk of dementia but also show structural and functional brain changes, particularly in regions vulnerable to cognitive impairment.^4^, ^5^

Although kidney dysfunction is linked to cognitive impairment, the relationship between cognitive function and estimated glomerular filtration rate (eGFR), the most widely used measure of kidney function, remains incompletely understood. Most existing studies focus on patients with CKD, whereas evidence from community‐dwelling populations without overt kidney disease is comparatively limited.^6^ It is therefore uncertain whether kidney‐brain associations observed in CKD are also present across the broader spectrum of kidney function in generally healthy individuals and whether the specific brain regions associated with kidney function are shared or differ between CKD and non‐CKD populations.

Additionally, prior studies largely relied on single time‐point assessments of kidney function.^7^ Longitudinal eGFR patterns may better capture biologically relevant kidney stress: Cumulative low eGFR reflects long‐term metabolic burden, while visit‐to‐visit variability reflects physiological instability. Both measures have been shown to predict cardiovascular events and mortality,^8^, ^9^ but their associations with cognitive function and brain structural changes remain largely unexplored.

To address these gaps, we analyzed data from three complementary cohorts, including two community‐based cohorts and one CKD cohort. We examined the associations of eGFR level and variability with cognitive function and brain structure. This cross‐cohort design enabled comparison of kidney‐brain associations across health and disease and identification of brain structural correlates of kidney function. We further investigated whether these associations are consistent across populations or population‐specific, providing insight into the neural correlates of kidney function and early markers of cognitive vulnerability.

**RESEARCH IN CONTEXT**

1. **Systematic review**: Existing evidence indicates that impaired kidney function is associated with cognitive decline and structural brain changes, particularly in patients with CKD. However, most studies relied on single time‐point measurements of eGFR. Longitudinal kidney function metrics, including cumulative exposure and visit‐to‐visit variability, have been less frequently investigated. In addition, little is known about how eGFR levels and variability relate to region‐specific brain structure or whether these associations are consistent across CKD and non‐CKD populations.
2. **Interpretation**: We integrated data from three complementary cohorts, including two community‐based cohorts (META‐KLS and UK Biobank) and one CKD cohort (BFH‐CKD), to examine the associations of kidney function with cognitive and brain structural outcomes. Lower eGFR levels and greater eGFR variability were associated with poorer cognitive function. Neuroimaging analyses further revealed that eGFR levels were associated with the WM, superior frontal, and middle temporal volumes across cohorts, whereas greater eGFR variability was linked to alterations in the inferior parietal, middle temporal, and rostral anterior cingulate cortex. These findings support a kidney‐brain axis and suggest that both kidney function and its stability may contribute to brain health.
3. **Future directions**: Future studies should explore the biological mechanisms linking dynamic kidney function to brain structure and cognitive trajectories. Interventional studies are also warranted to determine whether preserving kidney function and reducing its variability may help mitigate cognitive decline.

## 2 METHODS

### 2.1 Study population

This study included participants from three prospective cohorts: two community‐based cohorts, the Multimodality MEdical imaging sTudy bAsed on KaiLuan Study (META‐KLS) and the UK Biobank, and a hospital‐based cohort of patients with CKD, the Beijing Friendship Hospital Chronic Kidney Disease study (BFH‐CKD).

META‐KLS is a community‐based cohort nested within the Kailuan Study in Tangshan, Northern China. The Kailuan Study enrolled 101,510 adults aged ≥18 years between 2006 and 2007, with biennial follow‐up examinations. A total of 1195 participants underwent brain magnetic resonance imaging (MRI) in META‐KLS. For the present analysis, baseline was set at 2008 to ensure consistency in serum creatinine measurements.^10^ UK Biobank is a large population‐based cohort in the United Kingdom, which recruited over 500,000 participants aged 37 to 73 years between 2006 and 2010, with imaging data available in a subset from 2014.^11^, ^12^ The BFH‐CKD study is a hospital‐based cohort conducted at Beijing Friendship Hospital, including 490 CKD patients enrolled between 2022 and 2025.^13^ Eligible participants in BFH‐CKD were aged 18 to 70 years, right‐handed, and met established diagnostic criteria for CKD.^14^, ^15^ All cohorts were approved by their respective ethics committees.

A flow diagram of participant selection is presented in Figure S1. Across the three cohorts, participants with serum creatinine measurements and brain MRI data were included. Exclusions were mainly applied for poor‐quality MRI, extreme estimated total intracranial volume (eTIV), or a history of major neurological or systemic disease. Finally, 1019 participants from META‐KLS, 5828 from UK Biobank, and 426 from BFH‐CKD were included in our analysis.

### 2.2 Assessment and derivation of eGFR measures

Serum creatinine was measured six times in META‐KLS, twice in UK Biobank, and once in BFH‐CKD. eGFR was calculated from standardized serum creatinine using the 2021 race‐free Chronic Kidney Disease Epidemiology Collaboration (CKD‐EPI) creatinine equation,^16^ in accordance with Kidney Disease: Improving Global Outcomes (KDIGO) guidance.^17^

Because the availability of repeated measurements differed across cohorts, eGFR exposure was defined according to the data structure of each study. In META‐KLS, cumulative eGFR was calculated as the time‐weighted average of six measurements obtained between 2008 and 2020. In UK Biobank, eGFR exposure was defined as the mean of two available measurements, whereas in BFH‐CKD, baseline eGFR was used.

Visit‐to‐visit eGFR variability was assessed only in META‐KLS and quantified using variability independent of the mean (VIM), average real variability (ARV), standard deviation (SD), and coefficient of variation (CV). Higher eGFR levels indicate better kidney function, whereas higher variability reflects greater instability.

### 2.3 Assessment of cognitive function

Cognitive function was assessed using the Montreal Cognitive Assessment (MoCA) in META‐KLS and BFH‐CKD; no corresponding data were available in UK Biobank. The MoCA evaluates multiple cognitive domains, with total scores ranging from 0 to 30. Higher scores indicate better cognitive function.^18^ Assessments were administered face to face by trained personnel using standard procedures, with a one‐point adjustment for lower educational attainment. Scores were analyzed as continuous variables.

### 2.4 MRI acquisition, processing, and imaging‐derived phenotypes

Detailed MRI acquisition information, including scanner models and key T1‐weighted structural imaging parameters for each cohort, is provided in Table S1. For the UK Biobank cohort, we directly used pre‐computed brain imaging‐derived phenotypes.

To ensure comparability across cohorts, MRI data from META‐KLS and BFH‐CKD cohorts were processed using the same UK Biobank‐based neuroimaging framework (https://www.fmrib.ox.ac.uk/ukbiobank/). Subcortical structures were segmented using FSL‐FIRST implemented in FSL (version 6.0.6.4), and bilateral volumes were derived. Cortical reconstruction and parcellation were performed using FreeSurfer (version 7.3.2). Regional cortical measures, including cortical volume and thickness, were extracted according to the Desikan‐Killiany‐Tourville atlas.^19^

Neuroimaging outcomes included whole‐brain tissue volumes (gray matter [GM], white matter [WM], and total brain volume [GM+WM]), subcortical nuclei volumes, regional cortical volumes, and cortical thickness, which were categorized into prespecified neuroanatomical domains for analysis. Corresponding left‐ and right‐sided regions were merged to generate bilateral regional measures, resulting in 7 subcortical regions and 31 cortical regions for analysis. All measures underwent standardized quality control before analysis.

### 2.5 Statistical analysis

**FIGURE 1:**
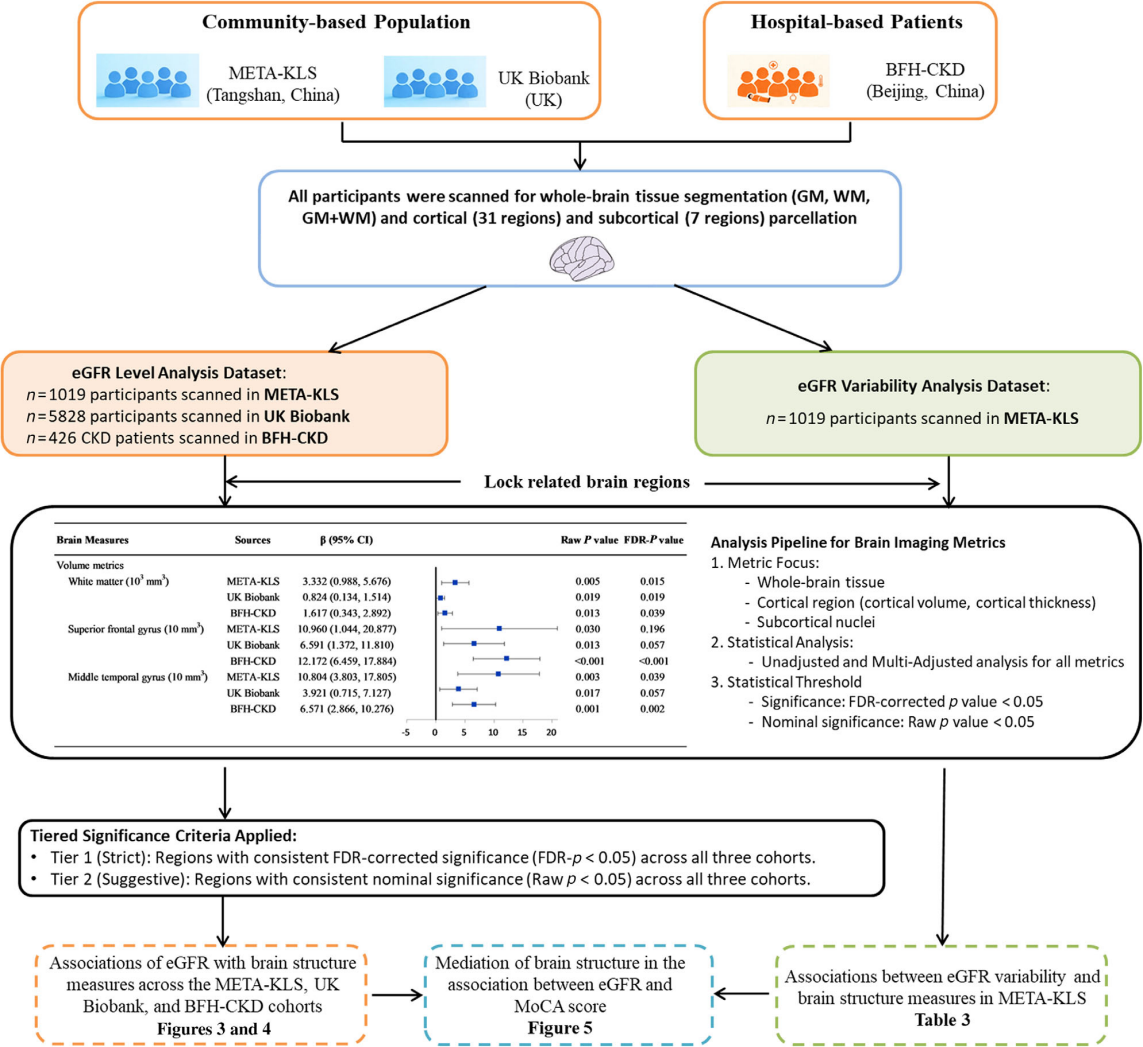
Study population and analytical workflow. META‐KLS, Multimodality MEdical imaging sTudy bAsed on Kailuan Study; BFH‐CKD study, Beijing Friendship Hospital Chronic Kidney Disease study; GM, gray matter; WM, white matter; eGFR, estimated glomerular filtration rate.

Analyses of eGFR level were performed across the META‐KLS, UK Biobank, and BFH‐CKD cohorts to identify shared and cohort‐specific brain structural correlates. Analyses of eGFR variability were restricted to the META‐KLS cohort, where repeated eGFR measurements were available. eGFR level was modeled per 10 mL/min/1.73 m^2^ increase, whereas eGFR variability metrics were analyzed per 1‐SD increase because no clinically meaningful unit has been established. The study population and analytical workflow are shown in Figure 1.

Associations between eGFR indicators with brain structural measures were assessed using univariate and multivariable linear regression models with a consistent covariate adjustment strategy across cohorts. Model 1 was unadjusted, where Model 2 adjusted for age, sex, education, smoking status, alcohol consumption, body mass index, hypertension, diabetes, triglycerides, high‐density lipoprotein cholesterol, and low‐density lipoprotein cholesterol. eTIV was additionally included in analyses of brain volume measures to account for differences in head size. Analyses of eGFR variability were further adjusted for baseline eGFR. The Benjamini–Hochberg false discovery rate (FDR) procedure was applied within each neuroanatomical domain to account for multiple testing. Cohort‐specific and shared associations were summarized using forest plots.

As an exploratory analysis, mediation models were conducted in the META‐KLS cohort to examine whether brain regions associated with eGFR across all three cohorts, as well as regions associated with eGFR variability in META‐KLS, mediated the association between kidney function and MoCA score. Direct and indirect effects were estimated using linear regression models, and the proportion mediated was calculated. All models were adjusted for prespecified covariates. Given the cross‐sectional nature of MRI and cognitive assessments, these mediation analyses were exploratory and do not imply causal mediation.

Missing covariate data in the UK Biobank and BFH‐CKD cohorts were handled using multiple imputation by chained equations. The imputation models included the eGFR exposure variables and all covariates used in the regression analyses. Twenty imputed datasets were generated and combined using Rubin's rules. Brain structural measures, as study outcomes, were not imputed.

All analyses were performed using SAS version 9.4 (SAS Institute, Cary, NC, USA) and R version 4.4.0 (R Foundation for Statistical Computing, Vienna, Austria).

## 3 RESULTS

### 3.1 Demographic and clinical characteristics

The baseline characteristics of 7273 participants across three cohorts are summarized in Table 1: 1019 in META‐KLS, 5828 in UK Biobank, and 426 in BFH‐CKD. When categorized by age, participants aged 45 to 65 years represented the largest proportion in META‐KLS and BFH‐CKD, whereas the proportion of participants aged over 65 years was highest in UK Biobank. Sex distribution differed across cohorts, with men accounting for 55.643% in META‐KLS, 49.914% in UK Biobank, and 65.258% in BFH‐CKD. The distribution of health behaviors and comorbidity profiles also varied. The proportion of current smokers ranged from 3.449% in UK Biobank to 47.399% in META‐KLS; the proportion of alcohol consumers ranged from 22.770% in BFH‐CKD to 94.252% in UK Biobank. Hypertension prevalence was 48.867% in META‐KLS, 51.132% in UK Biobank and 73.005% in BFH‐CKD; diabetes prevalence was 19.333% in META‐KLS, 4.358% in UK Biobank, and 34.272% in BFH‐CKD. Median eGFR ranged from 64.925 mL/min/1.73 m^2^ (interquartile range [IQR] 18.667, 93.665) in BFH‐CKD to 105.736 mL/min/1.73 m^2^ (IQR 96.130, 115.023) in META‐KLS.

**TABLE 1:** Demographic and clinical characteristics of study participants across three databases.

Characteristic	META‐KLS	UK Biobank^a^	BFH‐CKD^a^
No. of participants	1019	5828	426
Age, no. (%)			
≤45	266 (26.104)	29 (0.498)	143 (33.568)
45 to 65	448 (43.965)	2425 (41.609)	195 (45.775)
>65	305 (29.931)	3374 (57.893)	88 (20.657)
Male, no. (%)	567 (55.643)	2909 (49.914)	278 (65.258)
Higher education level,^b^ no. (%)	604 (59.274)	4824 (82.773)	321 (75.352)
Cumulative eGFR, median (IQR), mL/min/1.73 m^2^	105.736 (96.130, 115.023)	95.123 (86.791, 101.325)	64.925 (18.667, 93.665)
BMI, median (IQR), kg/m2	24.968 (23.253, 27.180)	25.861 (23.611, 28.680)	26.124 (23.292, 28.878)
Current smoker, no. (%)	483 (47.399)	201 (3.449)	125 (29.343)
Current alcohol consumption, no. (%)	655 (64.279)	5493 (94.252)	97 (22.770)
Blood pressure, median (IQR)			
Systolic, mmHg	132.667 (120.667, 145.333)	135.500 (124.500, 148.500)	135.000 (123.000, 150.000)
Diastolic, mmHg	80.667 (72.333, 88.000)	79.500 (73.500,86.500)	84.000 (76.000, 93.000)
History of hypertension, no. (%)	496 (48.867)	2980 (51.132)	311 (73.005)
History of diabetes, no. (%)	197 (19.333)	254 (4.358)	146 (34.272)
Cholesterol, median (IQR)			
Triglyceride, mmol/L	1.340 (0.930, 1.980)	1.430 (1.047, 2.012)	1.730 (1.230, 2.390)
High‐density lipoprotein, mmol/L	1.390 (1.180, 1.650)	1.483 (1.248, 1.784)	1.020 (0.840, 1.280)
Low‐density lipoprotein, mmol/L	3.090 (2.580, 3.600)	3.548 (2.985, 4.144)	2.780 (2.220, 3.690)
MoCA score, median (IQR)	25.000 (22.000, 27.000)	–	25.000 (23.000, 27.000)

Abbreviations: BFH‐CKD study, Beijing Friendship Hospital Chronic Kidney Disease study; BMI, body mass index; eGFR, estimated glomerular filtration rate; IQR, interquartile range; META‐KLS, Multimodality MEdical imaging sTudy bAsed on KaiLuan Study; MoCA, Montreal Cognitive Assessment; SD, standard deviation.

^a^ In UK Biobank, missing rates were 0.172% for education, 0.051% for BMI, 0.017% for alcohol consumption, 0.189% for smoking status, 0.635% for history of diabetes, 0.034% for triglycerides, 12.835% for HDL, and 0.137% for LDL. In BFH‐CKD, missing rates were 3.286% for BMI, 2.582% for blood pressure,4.930% for triglycerides, 9.155% for HDL, and 4.930% for LDL.

^b^ Higher education level was defined as > 9 years of schooling in the META‐KLS and BFH‐CKD cohorts, and as the attainment of Advanced levels (A levels) or higher qualifications (e.g., college/university degrees, professional diplomas) in the UK Biobank cohort.

### 3.2 Association of eGFR and cognitive function

Higher eGFR level was significantly associated with better MoCA performance (Table 2). In META‐KLS, compared with participants in the lowest tertile (Tertile 1), multivariable‐adjusted MoCA scores were higher among those in Tertile 2 (*β* = 0.796, 95% confidence interval [CI]: 0.273 to 1.319) and Tertile 3 (*β* = 0.747, 95% CI: 0.116 to 1.378). Consistently, when analyzed as a continuous variable, each 10 mL/min/1.73 m^2^ increase in cumulative eGFR was associated with a higher MoCA score (*β* = 0.350, 95% CI: 0.148 to 0.552). Similar associations were observed in BFH‐CKD, where higher eGFR was associated with higher MoCA scores across tertiles (Tertile 2: *β* = 0.954, 95% CI: 0.114 to 1.793; Tertile 3: *β* = 0.958, 95%CI: 0.078 to 1.837). When analyzed as a continuous variable, eGFR remained positively associated with MoCA performance, with each 10 mL/min/1.73 m^2^ increase linked to a higher MoCA score (*β* = 0.123, 95%CI: 0.030 to 0.217).

**TABLE 2:** Associations of eGFR levels and eGFR variability with MoCA scores.

		Tertiles of eGFR			
Variables	Continuous	Tertile 1	Tertile 2	Tertile 3	*P* for trend^a^
**eGFR levels (per 10 mL/min/1.73 m^2^ increase)** ^b^					
META‐KLS					
*β* (95% CI), Model 1^c^	1.218 (1.052, 1.382)*	Ref	2.010 (1.465, 2.555)*	3.631 (3.083, 4.179)*	<0.001
*β* (95% CI), Model 2^d^	0.350 (0.148, 0.552)*	Ref	0.796 (0.273, 1.319)*	0.747 (0.116, 1.378)*	0.015
BFH‐CKD					
*β* (95% CI), Model 1	0.187 (0.096, 0.279)*	Ref	0.968 (0.065, 1.871)*	1.428 (0.539, 2.317)*	0.002
*β* (95% CI), Model 2	0.123 (0.030, 0.217)*	Ref	0.954 (0.114, 1.793)*	0.958 (0.078, 1.837)*	0.038
**eGFR variability (per 1‐SD increase)**					
META‐KLS					
VIM					
*β* (95% CI), Model 1	−0.974 (−1.207, −0.741)*	Ref	−0.593 (−1.167, −0.019)*	−2.083 (−2.661, −1.506)*	<0.001
*β* (95% CI), Model 2	−0.454 (−0.659, −0.250)*	Ref	−0.026 (−0.518, 0.466)	−0.809 (−1.314, −0.304)*	0.002
ARV					
*β* (95% CI), Model 1	−0.424 (−0.663, −0.185)*	Ref	−0.338 (−0.920, 0.245)	−1.329 (−1.915, −0.743)*	<0.0001
β (95% CI), Model 2	−0.241 (−0.442, −0.040)*	Ref	0.016 (−0.474, 0.505)	−0.738 (−1.234, −0.243)*	0.004
SD					
β (95% CI), Model 1	−0.543 (−0.781, −0.304)*	Ref	−0.374 (−0.955, 0.208)	−1.445 (−2.030, −0.859)*	<0.001
β (95% CI), Model 2	−0.377 (−0.578, −0.176)*	Ref	−0.036 (−0.529, 0.458)	−0.806 (−1.303, −0.308)*	0.001
CV					
*β* (95% CI), Model 1	−0.807 (−1.042, −0.571)*	Ref	−0.651 (−1.230, −0.073)*	−1.745 (−2.327, −1.1626)*	<0.001
*β* (95% CI), Model 2	−0.422 (−0.624, −0.220)*	Ref	−0.231 (−0.725, 0.264)	−0.748 (−1.250, −0.246)*	0.004

Abbreviations: ARV, average real variability; BFH‐CKD Beijing Friendship Hospital Chronic Kidney Disease study; CI, confidence interval; CV, coefficient of variation; eGFR, estimated glomerular filtration rate; META‐KLS, Multimodality MEdical imaging sTudy bAsed on KaiLuan Study; MoCA, Montreal Cognitive Assessment; SD, standard deviation; VIM, variability independent of mean.

^a^ *P* for trend was calculated by modeling eGFR tertiles as a continuous variable.

^b^ The definition of eGFR levels varied by cohort data availability: Cumulative eGFR was used for the META‐KLS study (derived from six repeated longitudinal measurements), while cross‐sectional baseline eGFR was used for the BFH‐CKD study owing to single‐time data constraints.

^c^ Model 1 was unadjusted.

^d^ Model 2 was adjusted for age, sex, education, smoking status, alcohol consumption, body mass index, hypertension, diabetes, triglycerides, high‐density lipoprotein cholesterol, and low‐density lipoprotein cholesterol. For eGFR variability models, baseline eGFR was additionally adjusted.

*p < 0.050.

Higher eGFR variability was associated with poorer MoCA performance. In META‐KLS, each 1‐SD increase in eGFR variability (VIM) was associated with a lower MoCA score in the adjusted model (*β* = −0.454, 95% CI: −0.659 to −0.250). When analyzed by tertiles, MoCA scores declined monotonically with increasing eGFR variability, and participants in the highest tertile had significantly lower MoCA scores compared with the lowest tertile (*β* = −0.809, 95% CI: −1.314 to −0.304). Consistent results were observed when variability was quantified by ARV, SD, or CV.

### 3.3 Associations of eGFR level with whole‐brain and subcortical nucleus volumes

Higher eGFR was consistently associated with larger global brain volumes across cohorts. In META‐KLS, each 10 mL/min/1.73 m^2^ increase in cumulative eGFR was associated with larger total GM+WM volume (*β* = 4.900×10^3^ mm^3^, 95% CI: 1.167×10^3^ to 8.633×10^3^) and WM volume (*β* = 3.332×10^3^ mm^3^, 95% CI: 0.988×10^3^ to 5.676×10^3^) (Table S2). Similar associations were observed in UK Biobank, where higher mean eGFR was associated with larger total GM+WM, GM, and WM volumes (Table S3). In BFH‐CKD, higher baseline eGFR was associated with greater WM volume, whereas associations with total GM+WM and GM volumes were not significant (Table S4).

In addition, higher mean eGFR in UK Biobank was associated with larger caudate, putamen, hippocampus, thalamus, and accumbens volumes after FDR correction, whereas nominal associations with the thalamus and accumbens were observed in BFH‐CKD.

### 3.4 Associations of eGFR level with cortical volume

Higher eGFR was associated with greater cortical volume across multiple regions in all three cohorts. In META‐KLS, widespread associations observed in the unadjusted model were attenuated after multivariable adjustment, with only the inferior parietal lobule (*β* = 112.946 mm^3^, 95% CI: 40.934 to 184.958) and middle temporal gyrus (*β* = 108.042 mm^3^, 95% CI: 38.034 to 178.049) remaining significant after FDR correction. Associations involving the superior frontal gyrus, supramarginal gyrus, and precuneus remained nominal (Table S5).

In UK Biobank, only the fusiform gyrus (*β* = 33.486 mm^3^, 95% CI: 11.906 to 55.067) and entorhinal cortex (*β* = 12.086 mm^3^, 95% CI: 4.996 to 19.175) retained significance after multivariable adjustment and FDR correction, whereas several frontal, temporal, parietal, and cingulate regions showed nominal associations (Table S6).

By contrast, BFH‐CKD demonstrated more extensive cortical involvement. Higher baseline eGFR was associated with larger cortical volumes across frontal, parietal, temporal, occipital, and cingulate regions after FDR correction, with *β* coefficients ranging from 4.374 to 121.718 mm^3^ (Table S7).

### 3.5 Associations of eGFR level with cortical thickness

Associations between eGFR and cortical thickness were generally weaker than those observed for cortical volume. In both META‐KLS and UK Biobank, multiple regions showed nominal associations, but none remained significant after multivariable adjustment and FDR correction (Tables S8,S9). In contrast, BFH‐CKD showed significant positive associations across multiple frontal, parietal, temporal, cingulate, and insular regions after FDR correction, with *β* coefficients ranging from 3.359 to 8.570 µm (Table S10).

### 3.6 Shared and cohort‐specific brain structural associations

**FIGURE 2:**
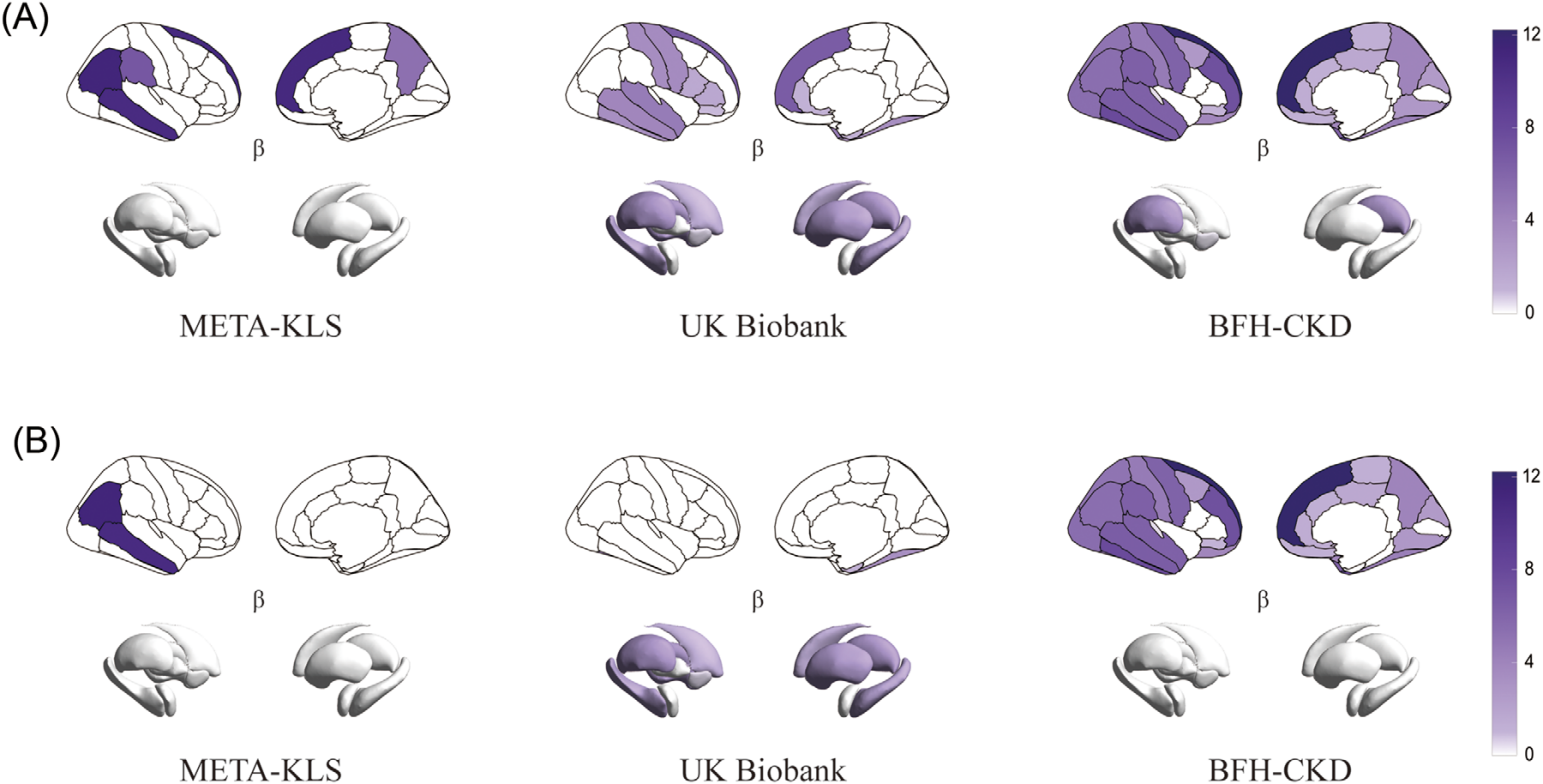
Cortical and subcortical volumes associated with eGFR levels in META‐KLS, UK Biobank, and BFH‐CKD cohorts. (A) Brain regions associated with eGFR levels at a nominal significance threshold (raw *p* < 0.05). (B) Regions remaining significant after false discovery rate correction (FDR‐*p* < 0.05). Colored regions indicate cortical and subcortical areas associated with eGFR levels. Darker shades indicate larger absolute *β* coefficients. eGFR, estimated glomerular filtration rate; BFH‐CKD, Beijing Friendship Hospital Chronic Kidney Disease study; META‐KLS, Multimodality MEdical imaging sTudy bAsed on Kailuan Study.

**FIGURE 3:**
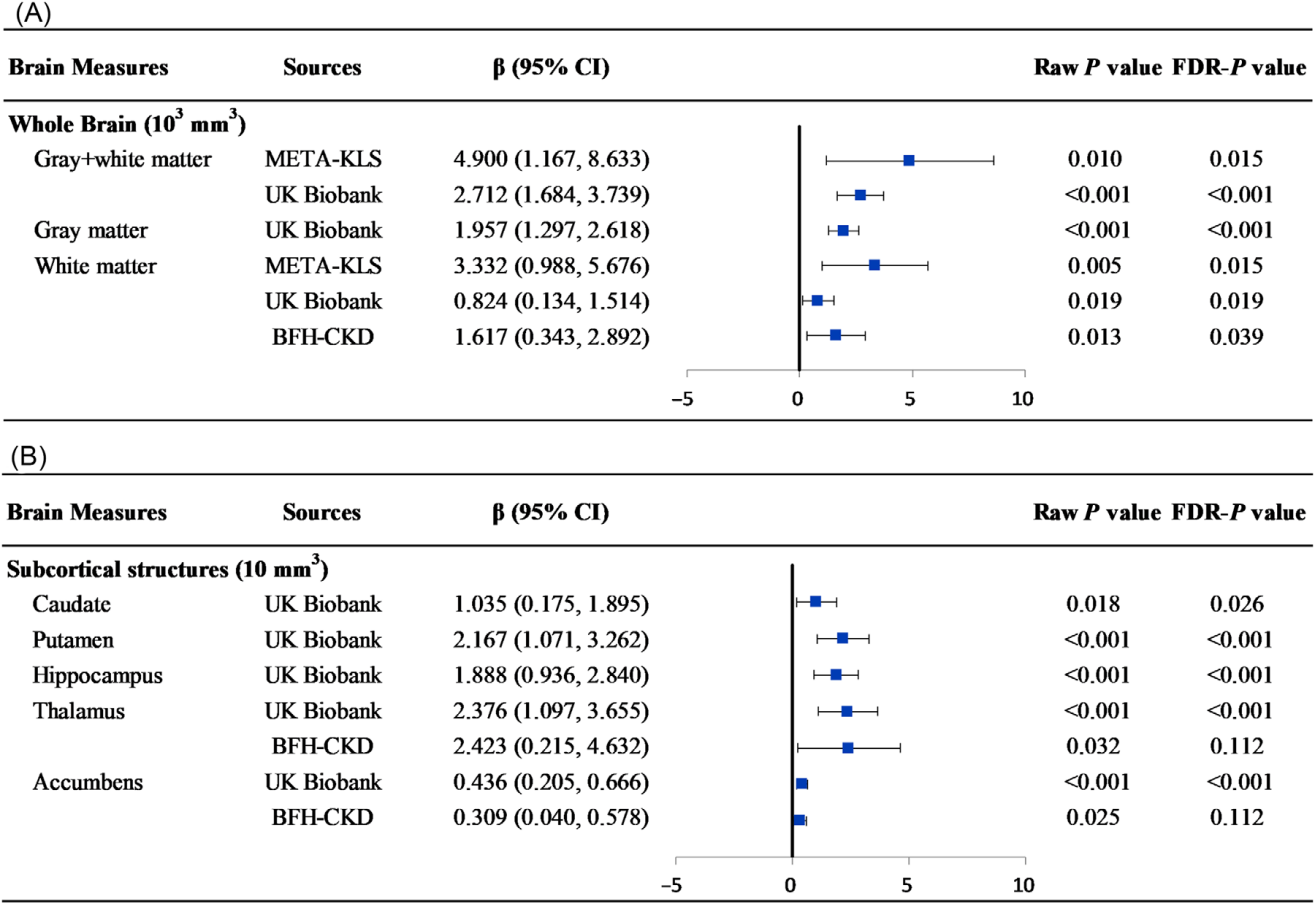
Associations of eGFR with whole‐brain and subcortical nuclei volume across the META‐KLS, UK Biobank, and BFH‐CKD cohorts. (A) Whole‐brain volume associations with eGFR. (B) Subcortical nucleus associations. Forest plot summarized cortical thickness with nominally (raw *p* < 0.05) or FDR‐significant association with eGFR in each cohort. *β* coefficients and 95% CIs were calculated after adjustment for age, sex, education, smoking status, alcohol consumption, body mass index, hypertension, diabetes, triglycerides, high‐density lipoprotein cholesterol, low‐density lipoprotein cholesterol and estimated total intracranial volume. Complete results for all whole‐brain and subcortical nuclei volume are provided in Tables S2,S3, and S4. eGFR, estimated glomerular filtration rate; CI, confidence interval.

**FIGURE 4:**
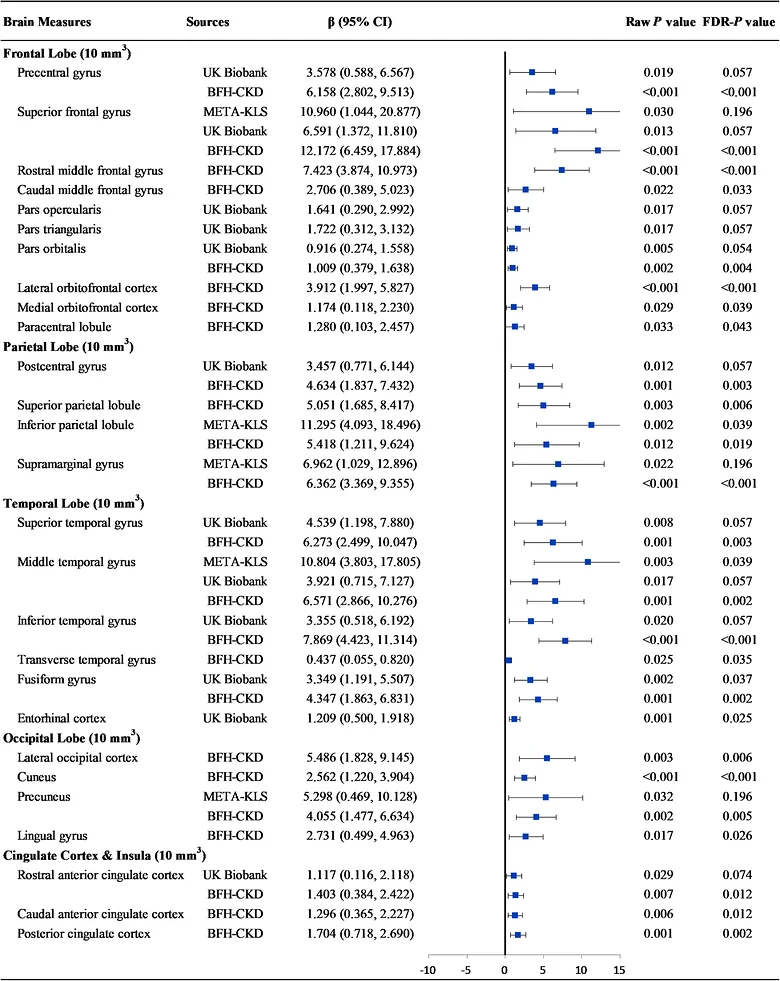
Associations of eGFR with cortical volume across META‐KLS, UK Biobank, and BFH‐CKD cohorts. Forest plot summarized cortical thickness with nominally (raw *p* < 0.05) or FDR‐significant association with eGFR in each cohort. *β* coefficients and 95% CIs were calculated after adjustment for age, sex, education, smoking status, alcohol consumption, body mass index, history of hypertension, history of diabetes, triglycerides, high‐density lipoprotein cholesterol, low‐density lipoprotein cholesterol, and estimated total intracranial volume. Complete results for all cortical volume are provided in Tables S5,S6, and S7. eGFR, estimated glomerular filtration rate; CI, confidence interval.

Cross‐cohort comparisons identified both shared and cohort‐specific brain structural associations (Figure 2). Positive associations of eGFR with the WM, superior frontal gyrus, and middle temporal gyrus volumes were consistently observed across all three cohorts (Figures 3, 4).

Pairwise comparisons revealed additional overlap. META‐KLS and UK Biobank shared a concordant association for total GM+WM volume. Concordant associations were also shared between UK Biobank and BFH‐CKD for the volumes of the thalamus, accumbens, precentral gyrus, pars orbitalis, postcentral gyrus, superior temporal gyrus, inferior temporal gyrus, fusiform gyrus, and rostral anterior cingulate cortex and between META‐KLS and BFH‐CKD for the volumes of the inferior parietal lobule, supramarginal gyrus, and precuneus, as well as the thicknesses of the postcentral and superior temporal gyri.

Despite these shared findings, each cohort also demonstrated distinct association patterns. BFH‐CKD showed the broadest cortical involvement, with associations spanning the frontal, parietal, temporal, occipital, and cingulate regions in both cortical volume and thickness analyses (Figure S2).

### 3.7 eGFR variability and brain structural measures in META‐KLS

After full adjustment, higher eGFR variability (per 1‐SD increase in VIM) was associated with smaller volume of the inferior parietal lobule (*β* = −84.372 mm^3^, 95% CI: −165.884 to −2.860) and middle temporal gyrus (*β* = −89.951 mm^3^, 95% CI: −169.071 to −10.831), as well as reduced thickness of the rostral anterior cingulate cortex (*β* = −9.035 µm, 95% CI: −17.470 to −0.600) (Table 3, Tables S11–S13). None of these associations remained significant after FDR correction.

**TABLE 3:** Associations between eGFR variability and brain structure measures in META‐KLS.

	Model 1^a^		Model 2^b^	
Brain Measures	*β* (95% CI)^c^	Raw *p* value	*β* (95% CI)	Raw *p* value
**Cortical volume (mm^3^)**				
Inferior parietal lobule				
VIM	−144.054 (−216.697, −71.411)	<0.001	−84.372 (−165.884, −2.860)	0.043
ARV	−49.975 (−123.012, 23.061)	0.180	−33.701 (−112.503, 45.102)	0.402
SD	−65.785 (−138.761, 7.190)	0.077	−52.248 (−127.906, 23.410)	0.176
CV	−113.35886 (−186.163, −40.555)	0.002	−70.547 (−149.050, 7.957)	0.078
Middle temporal gyrus				
VIM	−174.830 (−246.065, −103.595)	<0.001	−89.951 (−169.071, −10.831)	0.026
ARV	−72.165 (−143.954, −0.375)	0.049	−51.690 (−128.299, 24.920)	0.186
SD	−82.433 (−154.167, −10.699)	0.024	−63.569 (−137.009, 9.871)	0.090
CV	−138.425 (−209.899, −66.951)	<0.001	−78.612 (−154.811, −2.412)	0.043
**Cortical thickness (µm)**				
Rostral anterior cingulate cortex				
VIM	−9.489 (−16.965, −2.012)	0.013	−9.035 (−17.470, −0.600)	0.036
ARV	−0.912 (−8.411, 6.587)	0.811	−4.719 (−12.879, 3.442)	0.257
SD	−2.301 (−9.800, 5.199)	0.547	−5.814 (−13.650, 2.022)	0.146
CV	−6.636 (−14.124, 0.852)	0.082	−7.576 (−15.703, 0.551)	0.068

Abbreviations: ARV, average real variability; CI, confidence interval; CV, coefficient of variation; eGFR, estimated glomerular filtration rate; META‐KLS, Multimodality MEdical imaging sTudy bAsed on KaiLuan Study; SD, standard deviation; VIM, variability independent of mean.

^a^ Model 1 was unadjusted.

^b^ Model 2 was adjusted for age, sex, education, smoking status, alcohol consumption, body mass index, hypertension, diabetes, triglycerides, high‐density lipoprotein cholesterol, low‐density lipoprotein cholesterol, and baseline eGFR. Models for cortical volume were additionally adjusted for estimated total intracranial volume in both Models 1 and 2.

^c^ Regression coefficients (*β*) and 95% CIs were estimated per 1‐SD increase in each eGFR variability measure. Raw *p* values indicate unadjusted significance levels from the linear models.

Results based on ARV and SD showed similar directional trends but did not reach statistical significance. For CV, only the middle temporal gyrus showed a nominal association (*p* = 0.043).

### 3.8 Mediation of brain structure in the association between eGFR and cognitive function in META‐KLS

**FIGURE 5:**
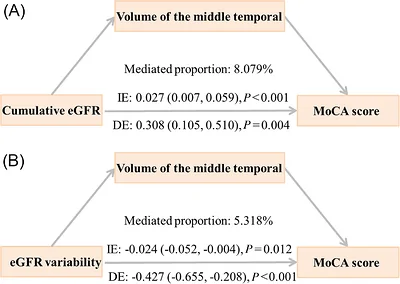
Mediation by middle temporal gyrus volume in associations of eGFR measures with MoCA score in META‐KLS. (A) Mediation analysis of cumulative eGFR. (B) Mediation analysis of eGFR variability. All models were adjusted for age, sex, education, smoking status, alcohol consumption, body mass index, hypertension, diabetes, triglycerides, high‐density lipoprotein cholesterol, low‐density lipoprotein cholesterol, and estimated total intracranial volume. For eGFR variability models, baseline eGFR was additionally adjusted. eGFR, estimated glomerular filtration rate; MoCA, Montreal Cognitive Assessment; DE, direct effect; IE, indirect effect.

Brain regions associated with eGFR level (WM volume, superior frontal gyrus, middle temporal gyrus) and eGFR variability (inferior parietal lobule, middle temporal gyrus, and rostral anterior cingulate cortex) were tested as potential mediators (Figures S3,S4). The volume of the middle temporal gyrus explained a small fraction of the associations of cumulative eGFR (8.079%) and eGFR variability (5.318%) with MoCA score. However, these findings were exploratory (Figure 5).

## 4 DISCUSSION

In this study integrating community‐based and CKD patient cohorts, kidney function was associated with both cognitive function and brain structure. Lower eGFR levels and greater eGFR variability were associated with poorer cognitive function. Kidney function also showed distinct patterns on brain structure. eGFR level was consistently associated with the volume of the WM, superior frontal gyrus, and middle temporal gyrus across cohorts. In contrast, higher eGFR variability was linked to regional structural alterations involving the inferior parietal lobule, middle temporal gyrus, and rostral anterior cingulate cortex. These findings support the concept of a kidney‐brain axis and suggest that both sustained kidney function and its variability may contribute to cognitive function and structural brain differences.

The association between eGFR level and cognitive function observed in this study is consistent with previous evidence indicating that preserved kidney function is important for maintaining brain health.^20^, ^21^ In both the META‐KLS and BFH‐CKD cohorts, lower eGFR level was associated with poorer MoCA performance, in line with findings from a large prospective CKD cohort showing that more advanced CKD severity is associated with a higher incidence of cognitive impairment over longitudinal follow‐up.^22^ Our findings extend these findings by demonstrating similar patterns across multiple cohorts with different population characteristics, thereby strengthening the generalizability of the observed relationship. Rather than reflecting only advanced kidney disease, these results suggest that even milder but sustained reductions in kidney function may be associated with poorer cognitive function.

Neuroimaging findings further support this interpretation. Lower eGFR was associated across cohorts with reduced WM volume and smaller volumes of the superior frontal gyrus and middle temporal gyrus. WM alterations may reflect broader disruption of brain structural integrity relevant to cognitive performance.^23^, ^24^ As a key temporal association cortex, the middle temporal gyrus is involved in semantic integration, memory‐related processing, and higher‐order cognition.^25^, ^26^ The superior frontal gyrus is considered part of the prefrontal networks supporting working memory and other higher‐order cognitive functions.^27^, ^28^ Together, these findings suggest that reduced kidney function may be related not only to diffuse brain atrophy but also to selective structural vulnerability in regions relevant to cognitive performance.^4^ In the META‐KLS cohort, mediation suggested that the volume of the middle temporal gyrus may represent a potential structural pathway linking cumulative kidney function burden to cognitive performance. Although the mediated proportion was modest, this finding provides exploratory neuroimaging evidence that regional cortical atrophy may be involved in the association between kidney function and cognition.^29^

Another contribution of this study is the demonstration that long‐term eGFR variability is associated with poorer cognitive function and alterations in regional brain structure. This finding suggests that kidney‐related brain risk may be captured not only by the cumulative burden of kidney dysfunction, but also by dynamic instability in kidney function over time. In the META‐KLS cohort, greater eGFR variability was associated with lower MoCA scores and more pronounced brain atrophy, predominantly in the inferior parietal lobule, middle temporal gyrus, and rostral anterior cingulate cortex. These observations extend prior work largely focused on single eGFR measurements or progressive decline and support the notion that visit‐to‐visit kidney function fluctuation may represent an additional pathway linking kidney dysfunction to brain injury. This distinction raises the possibility that individuals with similar mean eGFR levels may nonetheless differ in brain risk according to the stability of kidney function.

Several mechanisms may underlie the links between eGFR variability, brain structure, and cognitive performance. Chronic reduction in kidney function may promote cerebral injury through shared vascular risk factors, endothelial dysfunction, oxidative stress, inflammation, and accumulation of neurotoxic metabolites.^3^, ^30^ Superimposed fluctuations in eGFR may further aggravate these pathways, potentially through repeated hemodynamic instability, transient hypoperfusion‐reperfusion injury, episodic inflammatory activation, and fluctuating exposure to circulating uremic toxins.^31^, ^32^, ^33^ These pathophysiological processes may affect distributed cortical regions involved in cognition. Specifically, the inferior parietal lobule is implicated in attentional reorienting and working memory,^34^, ^35^ the middle temporal gyrus in semantic cognition,^36^ and the rostral anterior cingulate cortex in cognitive control and higher‐order regulatory processes.^37^ Mediation analysis for eGFR variability also suggested a modest indirect role of middle temporal gyrus volume in the association between kidney function instability and cognitive performance. Taken together with the findings for cumulative eGFR, the repeated involvement of the middle temporal gyrus suggests that the region may represent a regional substrate vulnerable to both the chronic burden and dynamic instability of kidney dysfunction.

This study has several strengths, including the integration of data from three independent cohorts (META‐KLS, UK Biobank, and BFH‐CKD). Nevertheless, several limitations should be acknowledged. First, differences in participant characteristics, recruitment strategies, follow‐up procedures, and MRI acquisition protocols across cohorts may have introduced heterogeneity. In addition, participants included in the MRI substudies of the UK Biobank and META‐KLS cohorts were generally healthier than the overall cohort populations, which may limit the generalizability of the neuroimaging findings. Second, although serum creatinine was measured using standardized IDMS‐traceable assays, minor measurement variability over time cannot be completely excluded. Third, although participants with recent initiation of medications known to affect kidney hemodynamics were excluded in the BFH‐CKD cohort, detailed longitudinal medication data were unavailable in the META‐KLS and UK Biobank cohorts. Fourth, cumulative eGFR exposure and eGFR variability could only be assessed in the META‐KLS cohort. Fifth, repeated urinary albumin‐to‐creatinine ratio measurements were unavailable or limited across cohorts, preventing assessment of longitudinal albuminuria burden and variability. Finally, because MRI and cognition were assessed concurrently in the META‐KLS cohort, the mediation findings should be considered exploratory rather than causal.

## AUTHOR CONTRIBUTIONS

Zhenchang Wang, Han Lv, Hao Wang conceived and designed the study. Li Yu, Lijun Song, Yanfang Tan, Xintian Ren were responsible for the data curation. Min Li, Wenbo Yang, Ying Hui performed the formal analysis and wrote the original draft. Huijing Shi, Yuntao Wu, Xiaoyan Bai, Xiaoshuai Li, Zhenghan Yang, Weikang Guo, Shouling Wu, Jing Li was responsible for project supervision. All authors reviewed the manuscript.

## CONFLICT OF INTEREST STATEMENT

The authors declare no conflicts of interest. Author disclosures are available in the Supporting Information.

## CONSENT STATEMENT

All human subjects provided informed consent.

## Supporting information




Supporting Information

Supporting Information

## Data Availability

UK Biobank data were accessed under application number 105978, and data access requests should be submitted directly to UK Biobank through its established application process. Data of the META‐KLS cohort and BFH‐CKD data are available upon approval by an independent review committee, with requests coordinated through the corresponding author, Dr. Hao Wang (wanghao4756@163.com).

## References

[1] Livingston G , Huntley J , Sommerlad A , et al. Dementia prevention, intervention, and care: 2020 report of the lancet commission. Lancet. 2020;396:413‐446. doi:10.1016/S0140-6736(20)30367-6 PMC7392084 32738937

[2] GBD 2019 Dementia Forecasting Collaborators . Estimation of the global prevalence of dementia in 2019 and forecasted prevalence in 2050: an analysis for the global burden of disease study 2019. Lancet Public Health. 2022;7:e105‐e125. doi:10.1016/S2468-2667(21)00249-8 PMC8810394 34998485

[3] Capasso G , Franssen CFM , Perna AF , et al. Drivers and mechanisms of cognitive decline in chronic kidney disease. Nat Rev Nephrol. 2025;21:536‐552. doi:10.1038/s41581-025-00963-0 PMC12279462 40281076

[4] Huang X , Yuan S , Ling Y , et al. Evaluating the effect of kidney function on brain volumes and dementia risk in the UK biobank. Arch Gerontol Geriatr. 2024;116:105157. doi:10.1016/j.archger.2023.105157 37634304

[5] Wang S , Wang J , Guo J , et al. Association of kidney function with dementia and structural brain differences: a large population‐based cohort study. J Gerontol A Biol Sci Med Sci. 2024;79:glad192. doi:10.1093/gerona/glad192 PMC10733178 37578935

[6] Huang Z , Yaffe K , Li C , et al. Chronic kidney disease severity and risk of cognitive impairment. JAMA Netw Open. 2026;9:e2559834. doi:10.1001/jamanetworkopen.2025.59834 PMC12914485 41701494

[7] Rahayel S , Goupil R , Genest DS , et al. Lower estimated glomerular filtration rate relates to cognitive impairment and brain alterations. Alzheimers Dement (Amst). 2024;16:e70044. doi:10.1002/dad2.70044 PMC11815220 39944593

[8] Al‐Aly Z , Balasubramanian S , McDonald JR , Scherrer JF , O'Hare AM . Greater variability in kidney function is associated with an increased risk of death. Kidney Int. 2012;82:1208‐1214. doi:10.1038/ki.2012.276 22854642

[9] Malhotra R , Katz R , Jotwani V , et al. Estimated GFR variability and risk of cardiovascular events and mortality in SPRINT (Systolic blood pressure intervention trial). Am J Kidney Dis. 2021;78:48‐56. doi:10.1053/j.ajkd.2020.10.016 PMC8200370 33333147

[10] Sun J , Hui Y , Li J , et al. Protocol for multi‐modality MEdical imaging sTudy bAsed on KaiLuan study (META‐KLS): rationale, design and database building. BMJ Open. 2023;13:e067283. doi:10.1136/bmjopen-2022-067283 PMC9923283 36764715

[11] Sudlow C , Gallacher J , Allen N , et al. UK biobank: an open access resource for identifying the causes of a wide range of complex diseases of middle and old age. PLoS Med. 2015;12:e1001779. doi:10.1371/journal.pmed.1001779 PMC4380465 25826379

[12] Alfaro‐Almagro F , Jenkinson M , Bangerter NK , et al. Image processing and quality control for the first 10,000 brain imaging datasets from UK biobank. Neuroimage. 2018;166:400‐424. doi:10.1016/j.neuroimage.2017.10.034 PMC5770339 29079522

[13] Song L , Wang H , Yang W , et al. Combination of rs‐fMRI, QSM, and ASL reveals the cerebral neurovascular coupling dysfunction is associated with cognitive decline in patients with chronic kidney disease. CNS Neurosci Ther. 2024;30:e70151. doi:10.1111/cns.70151 PMC11621384 39639681

[14] Inker LA , Astor BC , Fox CH , et al. KDOQI US commentary on the 2012 KDIGO clinical practice guideline for the evaluation and management of CKD. Am J Kidney Dis. 2014;63:713‐735. doi:10.1053/j.ajkd.2014.01.416 24647050

[15] Shlipak MG , Tummalapalli SL , Boulware LE , et al. The case for early identification and intervention of chronic kidney disease: conclusions from a kidney disease: improving global outcomes (KDIGO) controversies conference. Kidney Int. 2021;99:34‐47. doi:10.1016/j.kint.2020.10.012 33127436

[16] Inker LA , Eneanya ND , Coresh J , et al. New Creatinine‐ and Cystatin C‐Based equations to estimate GFR without race. N Engl J Med. 2021;385:1737‐1749. doi:10.1056/NEJMoa2102953 PMC8822996 34554658

[17] Kidney disease: improving global outcomes CKDWG. KDIGO 2024 clinical practice guideline for the evaluation and management of chronic kidney disease. Kidney Int. 2024;105:S117‐S314. doi:10.1016/j.kint.2023.10.018 38490803

[18] Nasreddine ZS , Phillips NA , Bedirian V , et al. The Montreal cognitive assessment, MoCA: a brief screening tool for mild cognitive impairment. J Am Geriatr Soc. 2005;53:695‐699. doi:10.1111/j.1532-5415.2005.53221.x 15817019

[19] Klein A , Tourville J . 101 labeled brain images and a consistent human cortical labeling protocol. Front Neurosci. 2012;6:171. doi:10.3389/fnins.2012.00171 PMC3514540 23227001

[20] Drew DA , Weiner DE , Sarnak MJ . Cognitive impairment in CKD: pathophysiology, management, and prevention. Am J Kidney Dis. 2019;74:782‐790. doi:10.1053/j.ajkd.2019.05.017 PMC7038648 31378643

[21] Xu H , Garcia‐Ptacek S , Trevisan M , et al. Kidney function, kidney function decline, and the risk of dementia in older adults: a registry‐based study. Neurology. 2021;96:e2956. doi:10.1212/WNL.0000000000012113 PMC8253567 33952656

[22] Anderer S . Chronic kidney disease severity linked with cognitive impairment risk. JAMA. 2026;335:1025. doi:10.1001/jama.2025.26406 41790461

[23] Madden DJ , Bennett IJ , Burzynska A , Potter GG , Chen NK , Song AW . Diffusion tensor imaging of cerebral white matter integrity in cognitive aging. Biochim Biophys Acta. 2012;1822:386‐400. doi:10.1016/j.bbadis.2011.08.003 PMC3241892 21871957

[24] He M , Yin Y , Qu J , et al. Deep learning for classifying and cognitive profiling of subcortical vascular cognitive impairment. Cyborg Bionic Syst. 2026;7:0561. doi:10.34133/cbsystems.0561 PMC13168761 42137062

[25] Binder JR , Desai RH , Graves WW , Conant LL . Where is the semantic system? A critical review and meta‐analysis of 120 functional neuroimaging studies. Cereb Cortex. 2009;19:2767‐2796. doi:10.1093/cercor/bhp055 PMC2774390 19329570

[26] Davey J , Thompson HE , Hallam G , et al. Exploring the role of the posterior middle temporal gyrus in semantic cognition: integration of anterior temporal lobe with executive processes. Neuroimage. 2016;137:165‐177. doi:10.1016/j.neuroimage.2016.05.051 PMC4927261 27236083

[27] du Boisgueheneuc F , Levy R , Volle E , et al. Functions of the left superior frontal gyrus in humans: a lesion study. Brain. 2006;129:3315‐3328. doi:10.1093/brain/awl244 16984899

[28] Friedman NP , Robbins TW . The role of prefrontal cortex in cognitive control and executive function. Neuropsychopharmacology. 2022;47:72‐89. doi:10.1038/s41386-021-01132-0 PMC8617292 34408280

[29] Wang X , Wang J , Xie Q , et al. Association between estimated glomerular filtration rate and memory function mediated by brain Gray matter volumes: a cross‐sectional study using the ADNI database. BMC Geriatr. 2025;25:628. doi:10.1186/s12877-025-06326-5 PMC12355792 40817206

[30] Viggiano D , Wagner CA , Martino G , et al. Mechanisms of cognitive dysfunction in CKD. Nat Rev Nephrol. 2020;16:452‐469. doi:10.1038/s41581-020-0266-9 32235904

[31] Nishiwaki H , Missikpode C , Ricardo AC , et al. Time‐Updated estimated GFR variability is associated with mortality, cardiovascular disease, and end‐stage kidney disease in patients with CKD: findings from the CRIC study. Am J Kidney Dis. 2025;85:695‐703 e1. doi:10.1053/j.ajkd.2025.01.010 PMC13143139 40054592

[32] Rajeev V , Chai YL , Poh L , et al. Chronic cerebral hypoperfusion: a critical feature in unravelling the etiology of vascular cognitive impairment. Acta Neuropathol Commun. 2023;11:93. doi:10.1186/s40478-023-01590-1 PMC10259064 37309012

[33] Glorieux G , Burtey S , Evenepoel P , et al. A guide to uraemic toxicity. Nat Rev Nephrol. 2026;22:50‐68. doi:10.1038/s41581-025-01006-4 40987897

[34] Krall SC , Rottschy C , Oberwelland E , et al. The role of the right temporoparietal junction in attention and social interaction as revealed by ALE meta‐analysis. Brain Struct Funct. 2015;220:587‐604. doi:10.1007/s00429-014-0803-z PMC4791048 24915964

[35] Berryhill ME , Chein J , Olson IR . At the intersection of attention and memory: the mechanistic role of the posterior parietal lobe in working memory. Neuropsychologia. 2011;49:1306‐1315. doi:10.1016/j.neuropsychologia.2011.02.033 PMC3078173 21345344

[36] Jackson RL . The neural correlates of semantic control revisited. Neuroimage. 2021;224:117444. doi:10.1016/j.neuroimage.2020.117444 PMC7779562 33059049

[37] Monosov IE , Haber SN , Leuthardt EC , Jezzini A . Anterior cingulate cortex and the control of dynamic behavior in primates. Curr Biol. 2020;30:R1442. doi:10.1016/j.cub.2020.10.009 PMC8197026 33290716

