# Supplementary material for: Kidney function and brain health: cognitive and structural signatures across cohorts
Source: Alzheimers Dement. 2026 Sep 23;22(9):e71840. doi: 10.1002/alz.71840 (PMC13598550; doi:10.1002/alz.71840)
Supplement: Supplementary file 2 [file ALZ-22-e71840-s001.docx]

**Kidney Function and Brain Health: Cognitive and Structural Signatures Across Cohorts**

Min Li ^1†^, Wenbo Yang ^2†^, Ying Hui ^3†^, Huijing Shi ^3^, Yuntao Wu ^3^, Li Yu ^2^, Lijun Song ^2^, Yanfang Tan ^1^, Xintian Ren ^1^, Xiaoyan Bai ^2^, Xiaoshuai Li ^2^, Zhenghan Yang ^2^, Weikang Guo ^4^, Shouling Wu ^5^, Jing Li ^6^, Zhenchang Wang ^2,7,8*^, Han Lv ^2,7,8*^, Hao Wang^2,7,8*^

^1^ Clinical Epidemiology and EBM Unit, Beijing Friendship Hospital, Capital Medical University, Beijing, China

^2^ Department of Radiology, Beijing Friendship Hospital, Capital Medical University, Beijing, China

^3^ Department of Radiology, Kailuan General Hospital, Tangshan, Hebei, China

^4^ Department of Nephrology, Beijing Friendship Hospital, Capital Medical University, Beijing, China

^5^ Department of Cardiology, Kailuan General Hospital, Tangshan, Hebei, China

^6^ Department of Radiology, Beijing Tsinghua Changgung Hospital, School of Clinical Medicine, Tsinghua University, China

^7^ Precision and Intelligence Medical Imaging Lab, Beijing Friendship Hospital, Capital Medical University, Beijing, China

^8^ Beijing Key Laboratory of Innovation and Transformation of Specialized CT/MR Imaging Equipment， Beijing, China

**Content**

**Table S1.** MRI Acquisition Information across Cohorts

**Table S2.** Associations of eGFR with Whole-Brain and Subcortical Volumes in META-KLS

**Table S3.** Associations of eGFR with Whole-Brain and Subcortical Volumes in UK Biobank

**Table S4.** Associations of eGFR with Whole-Brain and Subcortical Volumes in BFH-CKD

**Table S5.** Associations of eGFR with Cortical Volumes in META-KLS

**Table S6.** Associations of eGFR with Cortical Volumes in UK Biobank

**Table S7**. Associations of eGFR with Cortical Volumes in BFH-CKD

**Table S8.** Associations of eGFR with Cortical Thickness in META-KLS

**Table S9.** Associations of eGFR with Cortical Thickness in UK Biobank

**Table S10**. Associations of eGFR with Cortical Thickness in BFH-CKD

**Table S11**. Associations of eGFR Variability (VIM) with Whole-Brain and Subcortical Volumes in META-KLS

**Table S12**. Associations of eGFR Variability (VIM) and Cortical Volumes in META-KLS

**Table S13**. Associations of eGFR Variability (VIM) and Cortical Thickness in META-KLS

**Figure S1.** Flow Diagram of Participant Selection

**Figure S2**. Associations of eGFR with Cortical Thickness across the META-KLS, UK Biobank, and BFH-CKD Cohorts

**Figure S3**. Mediation Analysis of Brain Structures in the Association Between Cumulative eGFR and Cognitive Function in META-KLS

**Figure S4**. Mediation Analysis of Brain Structures in the Association Between eGFR Variability and Cognitive Function in META-KLS

**Table S1. MRI Acquisition Information across Cohorts**

| **Cohort** | **Site / data source** | **Scanner** | **T1-weighted structural MRI sequence and parameters** |
| --- | --- | --- | --- |
| UK Biobank | Multi-site brain MRI acquisition across UK Biobank imaging assessment centres. | MAGNETOM Skyra 3.0-T MRI scanner (Siemens Healthineers, Erlangen, Germany). | 3D MPRAGE, sagittal acquisition.TR, 2000ms; TI, 880ms; Voxel size, 1.0 × 1.0 × 1.0 mm; acquisition matrix, 208 × 256 × 256. [1] |
| META-KLS | Single-site brain MRI acquisition at Kailuan General Hospital, Tangshan, Hebei, China. | Discovery MR750W 3.0-T MR system(GE Healthcare, Milwaukee, WI, USA). | T1-weighted 3D BRAVO sequence. TR, 6.7ms; TE, 2.6 ms; flip angle, 15 degrees; slice thickness, 1 mm; FOV, 256 × 256 mm^2^; acquisition matrix, 256 × 256. [2] |
| BFH-CKD | Single-site brain MRI acquisition at Beijing Friendship Hospital, Beijing, China. | Discovery MR750W 3.0-T MR system (GE Healthcare, Milwaukee, WI, USA). | T1-weighted 3D BRAVO sequence. TR, 8.8 ms; TE, 3.5 ms; flip angle, 15 degrees; slice thickness, 1 mm; FOV, 240 × 240 mm^2^; acquisition matrix, 256 × 256. [3] |

Abbreviation: BRAVO, brain volume imaging; FOV, field of view; MPRAGE, magnetization-prepared rapid gradient echo; TE, echo time;TI, inversion time; TR, repetition time.

**Reference:**

[1] Miller KL, Alfaro-Almagro F, Bangerter NK, Thomas DL, Yacoub E, Xu J, et al. Multimodal population brain imaging in the UK Biobank prospective epidemiological study. *Nat Neurosci.* 2016;19(11):1523-1536. doi:10.1038/nn.4393.

[2] Sun J, Hui Y, Li J, Zhao X, Chen Q, Li X, et al. Protocol for Multi-modality MEdical imaging sTudy bAsed on KaiLuan Study (META-KLS): rationale, design and database building. *BMJ Open.* 2023;13(2):e067283. doi:10.1136/bmjopen-2022-067283.

[3] Wang H, Liu X, Song L, Yang W, Li M, Chen Q, et al. Dysfunctional coupling of cerebral blood flow and susceptibility value in the bilateral hippocampus is associated with cognitive decline in nondialysis patients with CKD. *J Am Soc Nephrol.* 2023;34(9):1574-1588. doi:10.1681/ASN.0000000000000185.

**Table S2. Associations of eGFR with Whole-Brain and Subcortical Volumes in META-KLS**

| Brain Measures | Model 1 ^1^ | |  | Model 2 ^2^ | |  |
| --- | --- | --- | --- | --- | --- | --- |
|  | β (95% CI) | P value |  | β (95% CI) | Raw *P* value | FDR-*P* value |
| Whole-brain metrics (10^3^ mm^3^) |  |  |  |  |  |  |
| Grey+white matter | 14.885 (12.047, 17.724) | <0.001 |  | 4.900 (1.167, 8.633) | 0.010 | 0.015 |
| Grey matter | 3.242 (1.652, 4.832) | <0.001 |  | 1.512 (-0.524, 3.548) | 0.145 | 0.145 |
| White matter | 11.566 (9.697, 13.436) | <0.001 |  | 3.332 (0.988, 5.676) | 0.005 | 0.015 |
| Subcortical structures (mm^3^) |  |  |  |  |  |  |
| Caudate | -13.453 (-70.046, 43.140) | 0.641 |  | -24.336 (-100.801, 52.130) | 0.532 | 0.730 |
| Putamen | 40.142 (-42.107, 122.392) | 0.338 |  | -15.980 (-127.026, 95.066) | 0.778 | 0.778 |
| Pallidum | -30.945 (-61.846, -0.044) | 0.050 |  | -30.214 (-71.385, 10.958) | 0.150 | 0.730 |
| Hippocampus | -18.397 (-76.518, 39.724) | 0.535 |  | -44.473 (-123.108, 34.161) | 0.267 | 0.730 |
| Amygdala | 0.715 (-23.912, 25.343) | 0.955 |  | -15.643 (-49.207, 17.921) | 0.361 | 0.730 |
| Thalamus | 27.761 (-110.414, 165.937) | 0.693 |  | -47.145 (-235.986, 141.695) | 0.624 | 0.730 |
| Accumbens | 8.918 (1.359, 16.476) | 0.021 |  | -2.529 (-12.707, 7.648) | 0.626 | 0.730 |

^1^ Model 1 was adjusted for estimated Total Intracranial Volume.

^2^ Model 2 was further adjusted for age, sex, education, smoking status, alcohol consumption, body mass index, hypertension, diabetes, triglycerides, high-density lipoprotein cholesterol, and low-density lipoprotein cholesterol. P-values in Model 2 were corrected for multiple comparisons using the False Discovery Rate (FDR) method; both raw P-values and FDR-corrected P-values (FDR-P) are presented.

Abbreviation: eGFR, estimated Glomerular Filtration Rate; META-KLS, Multimodality MEdical imaging sTudy bAsed on Kailuan Study; CI, Confidence Interval.

**Table S3. Associations of eGFR with Whole-Brain and Subcortical Volumes in UK Biobank**

| Brain Measures | Model 1 ^1^ | |  | Model 2 ^2^ | |  | Model 3 ^3^ | | |
| --- | --- | --- | --- | --- | --- | --- | --- | --- | --- |
|  | β (95% CI) | Raw *P* value |  | β (95% CI) | Raw *P* value |  | β (95% CI) | Raw *P* value | FDR-*P* value |
| Whole-brain metrics (10^3^ mm^3^) |  |  |  |  |  |  |  |  |  |
| Grey+white matter | 8.771 (7.738, 9.804) | <0.001 |  | 2.270 (1.158, 3.382) | <0.001 |  | 2.712 (1.684, 3.739) | <0.001 | <0.001 |
| Grey matter | 6.779 (6.093, 7.464) | <0.001 |  | 1.825 (1.110, 2.540) | <0.001 |  | 1.957 (1.297, 2.618) | <0.001 | <0.001 |
| White matter | 2.055 (1.406, 2.704) | <0.001 |  | 0.521 (-0.226, 1.269) | 0.171 |  | 0.824 (0.134, 1.514) | 0.019 | 0.019 |
| Subcortical structures (mm^3^) |  |  |  |  |  |  |  |  |  |
| Caudate | 22.508 (14.564, 30.451) | <0.001 |  | 9.896 (0.522, 19.270) | 0.039 |  | 10.349 (1.746, 18.951) | 0.018 | 0.026 |
| Putamen | 58.529 (48.027, 69.031) | <0.001 |  | 20.852 (8.878, 32.827) | 0.001 |  | 21.665 (10.713, 32.616) | <0.001 | <0.001 |
| Pallidum | 9.258 (4.832, 13.684) | <0.001 |  | 0.601 (-4.541, 5.744) | 0.819 |  | 1.693 (-3.043, 6.430) | 0.484 | 0.484 |
| Hippocampus | 45.704 (36.732, 54.676) | <0.001 |  | 16.888 (6.446, 27.330) | 0.002 |  | 18.880 (9.356, 28.404) | <0.001 | <0.001 |
| Amygdala | -4.487 (-9.222, 0.248) | 0.063 |  | -3.142 (-8.731, 2.447) | 0.271 |  | -2.087 (-7.199, 3.026) | 0.424 | 0.484 |
| Thalamus | 83.946 (71.468, 96.424) | <0.001 |  | 20.786 (6.696, 34.875) | 0.004 |  | 23.759 (10.965, 36.553) | <0.001 | <0.001 |
| Accumbens | 16.389 (14.135, 18.643) | <0.001 |  | 4.044 (1.522, 6.566) | 0.002 |  | 4.356 (2.054, 6.657) | <0.001 | <0.001 |

^1^ Model 1 was adjusted for estimated Total Intracranial Volume.

^2^ Model 2 was further adjusted for age, sex, education, smoking status, alcohol consumption, body mass index, hypertension, diabetes, triglycerides, high-density lipoprotein cholesterol, and low-density lipoprotein cholesterol.

^3^ Model 3 was based on the covariates in Model 2, with missing data handled via multiple imputations. P-values in Model 3 were corrected for multiple comparisons using the False Discovery Rate (FDR) method; both raw P-values and FDR-corrected P-values (FDR-P) are presented.

Abbreviation: eGFR, estimated Glomerular Filtration Rate; CI, Confidence Interval.

**Table S4. Associations of eGFR with Whole-Brain and Subcortical Volumes in BFH-CKD**

| Brain Measures | Model 1 ^1^ | |  | Model 2 ^2^ | |  | Model 3 ^3^ | | |
| --- | --- | --- | --- | --- | --- | --- | --- | --- | --- |
|  | β (95% CI) | Raw *P* value |  | β (95% CI) | Raw *P* value |  | β (95% CI) | Raw *P* value | FDR-*P* value |
| Whole-brain metrics (10^3^ mm^3^) |  |  |  |  |  |  |  |  |  |
| Grey+white matter | 4.723 (2.692, 6.755) | <0.001 |  | 2.268 (0.0144, 4.523) | 0.049 |  | 2.006 (-0.106, 4.118) | 0.063 | 0.095 |
| Grey matter | 1.403 (0.518, 2.288) | 0.002 |  | 0.456 (-0.561, 1.472) | 0.378 |  | 0.352 (-0.586, 1.289) | 0.462 | 0.462 |
| White matter | 3.318 (2.086, 4.550) | <0.001 |  | 1.772 (0.424, 3.119) | 0.010 |  | 1.617 (0.343, 2.892) | 0.013 | 0.039 |
| Subcortical structures (mm^3^) |  |  |  |  |  |  |  |  |  |
| Caudate | 10.173 (-1.699, 22.046) | 0.093 |  | 2.234 (-12.022, 16.490) | 0.758 |  | -0.410 (-13.418, 12.598) | 0.951 | 0.995 |
| Putamen | 19.174 (3.425, 34.922) | 0.017 |  | 24.861 (5.880, 43.842) | 0.010 |  | 15.052 (-1.924, 32.029) | 0.082 | 0.191 |
| Pallidum | 2.474 (-3.629, 8.576) | 0.426 |  | 0.714, (-6.489, 7.917) | 0.846 |  | 1.046 (-5.665, 7.756) | 0.760 | 0.995 |
| Hippocampus | 8.229 (-2.399, 18.856) | 0.129 |  | 2.925 (-9.900, 15.750) | 0.654 |  | -0.037 (-11.887, 11.812) | 0.995 | 0.995 |
| Amygdala | 7.005 (1.3129, 12.697) | 0.016 |  | 4.891 (-1.997, 11.779) | 0.163 |  | 3.178 (-3.241, 9.597) | 0.332 | 0.581 |
| Thalamus | 42.862 (22.724, 63.001) | <0.001 |  | 33.404 (9.097, 57.711) | 0.007 |  | 24.234 (2.154, 46.315) | 0.032 | 0.112 |
| Accumbens | 5.361 (2.921, 7.802) | <0.001 |  | 4.070 (1.1894, 6.951) | 0.006 |  | 3.087 (0.397, 5.777) | 0.025 | 0.112 |

^1^ Model 1 was adjusted for estimated Total Intracranial Volume.

^2^ Model 2 was further adjusted for age, sex, education, smoking status, alcohol consumption, body mass index, hypertension, diabetes, triglycerides, high-density lipoprotein cholesterol, and low-density lipoprotein cholesterol.

^3^ Model 3 was based on the covariates in Model 2, with missing data handled via multiple imputations. P-values in Model 3 were corrected for multiple comparisons using the False Discovery Rate (FDR) method; both raw P-values and FDR-corrected P-values (FDR-P) are presented.

Abbreviation: eGFR, estimated Glomerular Filtration Rate; CI, Confidence Interval; BFH-CKD study, Beijing Friendship Hospital Chronic Kidney Disease study.

**Table S5. Associations of eGFR with Cortical Volumes in META-KLS**

| Brain Measures | Model 1 ^1^ | |  | Model 2 ^2^ | |  |
| --- | --- | --- | --- | --- | --- | --- |
|  | β (95% CI) | P value |  | β (95% CI) | Raw *P* value | FDR-*P* value |
| Frontal Lobe (mm^3^) |  |  |  |  |  |  |
| Precentral gyrus | 104.324 (57.256, 151.392) | <0.001 |  | -4.491 (-67.355, 58.372) | 0.889 | 0.992 |
| Superior frontal gyrus | 292.107 (217.628, 366.585) | <0.001 |  | 109.604 (10.443, 208.765) | 0.030 | 0.196 |
| Rostral middle frontal gyrus | 119.258 (68.495, 170.021) | <0.001 |  | 14.087 (-54.478, 82.652) | 0.687 | 0.926 |
| Caudal middle frontal gyrus | 91.236 (56.845, 125.628) | <0.001 |  | 43.390 (-3.117, 89.897) | 0.067 | 0.282 |
| Pars opercularis | 53.309 (30.038, 76.579) | <0.001 |  | 19.741 (-11.500, 50.983) | 0.215 | 0.477 |
| Pars triangularis | 28.676 (4.472, 52.879) | 0.020 |  | 1.215 (-31.525, 33.954) | 0.942 | 0.992 |
| Pars orbitalis | 16.244 (5.881, 26.608) | 0.002 |  | 0.903 (-13.122, 14.927) | 0.900 | 0.992 |
| Lateral orbitofrontal cortex | 36.999 (8.429, 65.569) | 0.011 |  | -0.787 (-39.736, 38.162) | 0.968 | 0.992 |
| Medial orbitofrontal cortex | 45.872 (31.561, 60.183) | <0.001 |  | 16.317 (-2.975, 35.608) | 0.097 | 0.335 |
| Paracentral lobule | 37.531 (20.829, 54.233) | <0.001 |  | 11.040 (-11.405, 33.485) | 0.335 | 0.582 |
| Parietal Lobe (mm^3^) |  |  |  |  |  |  |
| Postcentral gyrus | 47.309 (7.358, 87.259) | 0.020 |  | -6.455 (-60.524, 47.614) | 0.815 | 0.992 |
| Superior parietal lobule | 89.913 (41.743, 138.082) | <0.001 |  | 47.274 (-17.329, 111.877) | 0.151 | 0.401 |
| Inferior parietal lobule | 209.135 (155.514, 262.757) | <0.001 |  | 112.946 (40.934, 184.958) | 0.002 | 0.039 |
| Supramarginal gyrus | 84.822 (40.910, 128.735) | <0.001 |  | 69.623 (10.290, 128.957) | 0.022 | 0.196 |
| Temporal Lobe (mm^3^) |  |  |  |  |  |  |
| Superior temporal gyrus | 141.477 (88.124, 194.829) | <0.001 |  | 52.205 (-19.844, 124.254) | 0.155 | 0.401 |
| Middle temporal gyrus | 244.767 (192.539, 296.996) | <0.001 |  | 108.042 (38.034, 178.049) | 0.003 | 0.039 |
| Inferior temporal gyrus | 63.594 (10.749, 116.439) | 0.018 |  | 19.426 (-52.510, 91.361) | 0.596 | 0.898 |
| Transverse temporal gyrus | 11.841 (5.926, 17.756) | <0.001 |  | -0.752 (-8.714, 7.210) | 0.853 | 0.992 |
| Fusiform gyrus | 71.200 (38.139, 104.262) | <0.001 |  | 40.459 (-3.741, 84.660) | 0.073 | 0.282 |
| Entorhinal cortex | 3.033 (-8.716, 14.782) | 0.613 |  | 6.691 (-9.249, 22.631) | 0.410 | 0.669 |
| Parahippocampal gyrus | 22.937 (12.422, 33.452) | <0.001 |  | 3.740 (-10.581, 18.060) | 0.609 | 0.898 |
| Occipital Lobe (mm^3^) |  |  |  |  |  |  |
| Lateral occipital cortex | 51.983 (2.524, 101.442) | 0.039 |  | 0.364 (-66.997, 67.725) | 0.992 | 0.992 |
| Cuneus | 12.239 (-8.299, 32.777) | 0.243 |  | -16.649 (-44.529, 11.231) | 0.242 | 0.499 |
| Precuneus | 93.863 (58.044, 129.683) | <0.001 |  | 52.984 (4.691, 101.278) | 0.032 | 0.196 |
| Lingual gyrus | 18.389 (-17.639, 54.416) | 0.317 |  | -35.444 (-84.077, 13.189) | 0.153 | 0.401 |
| Pericalcarine cortex | -15.371 (-35.348, 4.606) | 0.131 |  | -25.702 (-52.939, 1.536) | 0.064 | 0.282 |
| Cingulate Cortex & Insula (mm^3^) |  |  |  |  |  |  |
| Rostral anterior cingulate cortex | 25.305 (9.813, 40.797) | 0.001 |  | 13.608 (-7.478, 34.693) | 0.206 | 0.477 |
| Caudal anterior cingulate cortex | 31.657 (16.779, 46.536) | <0.001 |  | 4.511 (-15.503, 24.525) | 0.658 | 0.926 |
| Posterior cingulate cortex | 66.640 (47.475, 85.805) | <0.001 |  | 13.863 (-11.733, 39.460) | 0.288 | 0.558 |
| Isthmus of cingulate cortex | 14.679 (2.357, 27.001) | 0.020 |  | 8.086 (-8.473, 24.644) | 0.338 | 0.582 |
| Insula | 13.660 (-5.883, 33.203) | 0.171 |  | 4.828 (-21.318, 30.974) | 0.717 | 0.926 |

^1^ Model 1 was adjusted for estimated Total Intracranial Volume.

^2^ Model 2 was further adjusted for age, sex, education, smoking status, alcohol consumption, body mass index, hypertension, diabetes, triglycerides, high-density lipoprotein cholesterol, and low-density lipoprotein cholesterol. P-values in Model 2 were corrected for multiple comparisons using the False Discovery Rate (FDR) method; both raw P-values and FDR-corrected P-values (FDR-P) are presented.

Abbreviation: eGFR, estimated Glomerular Filtration Rate; META-KLS, Multimodality MEdical imaging sTudy bAsed on Kailuan Study; CI, Confidence Interval.

**Table S6. Associations of eGFR with Cortical Volumes in UK Biobank**

| Brain Measures | Model 1 ^1^ | |  | Model 2 ^2^ | |  | Model 3 ^3^ | | |
| --- | --- | --- | --- | --- | --- | --- | --- | --- | --- |
|  | β (95% CI) | Raw *P* value |  | β (95% CI) | Raw *P* value |  | β (95% CI) | Raw *P* value | FDR-*P* value |
| Frontal Lobe (mm^3^) |  |  |  |  |  |  |  |  |  |
| Precentral gyrus | 109.922 (81.919, 137.924) | <0.001 |  | 31.400 (-1.135, 63.935) | 0.059 |  | 35.775 (5.879, 65.670) | 0.019 | 0.057 |
| Superior frontal gyrus | 226.050 (176.542, 275.558) | <0.001 |  | 60.227 (3.312, 117.142) | 0.038 |  | 65.910 (13.716, 118.104) | 0.013 | 0.057 |
| Rostral middle frontal gyrus | 74.475 (45.667, 103.284) | <0.001 |  | -6.365 (-39.872, 27.141) | 0.710 |  | -4.619 (-35.299, 26.061) | 0.768 | 0.821 |
| Caudal middle frontal gyrus | 48.143 (29.321, 66.964) | <0.001 |  | -3.398 (-25.333, 18.538) | 0.761 |  | 1.746 (-18.440, 21.932) | 0.865 | 0.894 |
| Pars opercularis | 42.872 (30.298, 55.445) | <0.001 |  | 18.554 (3.901, 33.207) | 0.013 |  | 16.412 (2.900, 29.924) | 0.017 | 0.057 |
| Pars triangularis | 42.451 (29.287, 55.615) | <0.001 |  | 20.266 (4.943, 35.588) | 0.010 |  | 17.221 (3.119, 31.322) | 0.017 | 0.057 |
| Pars orbitalis | 21.451 (15.425, 27.478) | <0.001 |  | 8.595 (1.591, 15.598) | 0.016 |  | 9.160 (2.738, 15.581) | 0.005 | 0.054 |
| Lateral orbitofrontal cortex | 45.702 (29.592, 61.813) | <0.001 |  | 6.966 (-11.813, 25.745) | 0.467 |  | 8.143 (-9.060, 25.347) | 0.354 | 0.467 |
| Medial orbitofrontal cortex | 29.465 (19.427, 39.502) | <0.001 |  | 9.537 (-2.220, 21.294) | 0.112 |  | 9.096 (-1.664, 19.857) | 0.098 | 0.207 |
| Paracentral lobule | 32.790 (22.190, 43.390) | <0.001 |  | 8.992 (-3.315, 21.299) | 0.152 |  | 8.439 (-2.958, 19.837) | 0.147 | 0.253 |
| Parietal Lobe (mm^3^) |  |  |  |  |  |  |  |  |  |
| Postcentral gyrus | 85.613 (60.687, 110.538) | <0.001 |  | 35.085 (5.825, 64.346) | 0.019 |  | 34.573 (7.710, 61.435) | 0.012 | 0.057 |
| Superior parietal lobule | 78.189 (49.813, 106.564) | <0.001 |  | 4.647 (-28.473, 37.767) | 0.783 |  | 14.215 (-16.302, 44.732) | 0.361 | 0.467 |
| Inferior parietal lobule | 115.406 (81.645, 149.166) | <0.001 |  | 28.256 (-11.220, 67.732) | 0.161 |  | 29.195 (-6.952, 65.341) | 0.113 | 0.207 |
| Supramarginal gyrus | 55.338 (29.546, 81.131) | <0.001 |  | 5.653 (-24.700, 36.006) | 0.715 |  | 10.437 (-17.361, 38.235) | 0.462 | 0.573 |
| Temporal Lobe (mm^3^) |  |  |  |  |  |  |  |  |  |
| Superior temporal gyrus | 117.911 (86.685, 149.137) | <0.001 |  | 32.553 (-3.835, 68.941) | 0.080 |  | 45.391 (11.978, 78.804) | 0.008 | 0.057 |
| Middle temporal gyrus | 115.238 (85.025, 145.450) | <0.001 |  | 26.266 (-8.752, 61.284) | 0.142 |  | 39.209 (7.147, 71.270) | 0.017 | 0.057 |
| Inferior temporal gyrus | 77.452 (50.955, 103.949) | <0.001 |  | 36.282 (5.388, 67.176) | 0.021 |  | 33.552 (5.183, 61.921) | 0.020 | 0.057 |
| Transverse temporal gyrus | 5.591 (1.854, 9.328) | 0.003 |  | 2.798 (-1.617, 7.213) | 0.214 |  | 3.606 (-0.452 7.665) | 0.082 | 0.194 |
| Fusiform gyrus | 75.314 (55.039, 95.588) | <0.001 |  | 30.351 (6.681, 54.022) | 0.012 |  | 33.486 (11.906, 55.067) | 0.002 | 0.037 |
| Entorhinal cortex | 9.117 (2.569, 15.665) | 0.006 |  | 9.849 (2.092, 17.607) | 0.013 |  | 12.086 (4.996, 19.175) | 0.001 | 0.025 |
| Parahippocampal gyrus | 18.735 (12.794, 24.677) | <0.001 |  | 2.801 (-4.141, 9.743) | 0.429 |  | 5.290 (-1.098, 11.679) | 0.105 | 0.207 |
| Occipital Lobe (mm^3^) |  |  |  |  |  |  |  |  |  |
| Lateral occipital cortex | 61.961 (31.754, 92.167) | <0.001 |  | 22.5199 (-12.333, 57.373) | 0.205 |  | 25.910 (-6.169, 57.990) | 0.113 | 0.207 |
| Cuneus | 9.534 (-3.252, 22.321) | 0.144 |  | 0.774 (-14.225, 15.773) | 0.919 |  | 2.770 (-11.036, 16.577) | 0.694 | 0.780 |
| Precuneus | 72.927 (50.297, 95.558) | <0.001 |  | 9.734 (-16.610, 36.078) | 0.469 |  | 12.886 (-11.351, 37.122) | 0.297 | 0.439 |
| Lingual gyrus | 11.276 (-10.196, 32.748) | 0.303 |  | -15.766 (-41.043, 9.512) | 0.222 |  | -14.856 (-38.089, 8.378) | 0.210 | 0.326 |
| Pericalcarine cortex | -6.247 (-15.502, 3.009) | 0.186 |  | -3.756 (-14.665, 7.154) | 0.500 |  | -1.948 (-12.008, 8.111) | 0.704 | 0.780 |
| Cingulate Cortex & Insula (mm^3^) |  |  |  |  |  |  |  |  |  |
| Rostral anterior cingulate cortex | 20.001 (10.742, 29.260) | <0.001 |  | 8.361 (-2.549, 19.271) | 0.133 |  | 11.169 (1.162, 21.176) | 0.029 | 0.074 |
| Caudal anterior cingulate cortex | 15.105 (5.794, 24.415) | 0.002 |  | -2.236 (-13.283, 8.810) | 0.692 |  | -0.613 (-10.684, 9.458) | 0.905 | 0.905 |
| Posterior cingulate cortex | 20.161 (11.224, 29.099) | <0.001 |  | 3.542 (-6.955, 14.039) | 0.508 |  | 6.347 (-3.307, 16.001) | 0.198 | 0.322 |
| Isthmus of cingulate cortex | 2.090 (-4.873, 9.054) | 0.556 |  | 1.988 (-6.170, 10.147) | 0.633 |  | 2.492 (-5.016, 10.001) | 0.515 | 0.614 |
| Insula | 0.636 (-11.075, 12.347) | 0.915 |  | 3.033 (-10.567, 16.634) | 0.662 |  | 5.858 (-6.715, 18.430) | 0.361 | 0.467 |

^1^ Model 1 was adjusted for estimated Total Intracranial Volume.

^2^ Model 2 was further adjusted for age, sex, education, smoking status, alcohol consumption, body mass index, hypertension, diabetes, triglycerides, high-density lipoprotein cholesterol, and low-density lipoprotein cholesterol.

^3^ Model 3 was based on the covariates in Model 2, with missing data handled via multiple imputations. P-values in Model 3 were corrected for multiple comparisons using the False Discovery Rate (FDR) method; both raw P-values and FDR-corrected P-values (FDR-P) are presented.

Abbreviation: eGFR, estimated Glomerular Filtration Rate; CI, Confidence Interval.

**Table S7. Associations of eGFR with Cortical Volumes in BFH-CKD**

| Brain Measures | Model 1 ^1^ | |  | Model 2 ^2^ | |  | Model 3 ^3^ | | |
| --- | --- | --- | --- | --- | --- | --- | --- | --- | --- |
|  | β (95% CI) | Raw *P* value |  | β (95% CI) | Raw *P* value |  | β (95% CI) | Raw *P* value | FDR-*P* value |
| Frontal Lobe (mm^3^) |  |  |  |  |  |  |  |  |  |
| Precentral gyrus | 90.608 (60.143, 121.073) | <0.001 |  | 57.406 (20.423, 94.388) | 0.002 |  | 61.576 (28.017, 95.134) | <0.001 | <0.001 |
| Superior frontal gyrus | 187.930 (132.668, 243.192) | <0.001 |  | 113.387 (51.358, 175.415) | <0.001 |  | 121.718 (64.592, 178.843) | <0.001 | <0.001 |
| Rostral middle frontal gyrus | 102.005 (67.651, 136.358) | <0.001 |  | 80.692 (41.538, 119.847) | <0.001 |  | 74.234 (38.741, 109.728) | <0.001 | <0.001 |
| Caudal middle frontal gyrus | 40.870 (20.154, 61.587) | <0.001 |  | 24.033 (-0.722, 48.787) | 0.057 |  | 27.057 (3.888, 50.227) | 0.022 | 0.033 |
| Pars opercularis | 27.074 (12.081, 42.068) | <0.001 |  | 17.333 (-0.620, 35.286) | 0.058 |  | 13.759 (-2.673, 30.191) | 0.101 | 0.120 |
| Pars triangularis | 29.548 (14.246, 44.850) | <0.001 |  | 12.839 (-5.404, 31.083) | 0.167 |  | 12.933 (-3.816, 29.683) | 0.130 | 0.149 |
| Pars orbitalis | 17.919 (12.050, 23.788) | <0.001 |  | 9.646 (2.859, 16.434) | 0.006 |  | 10.088 (3.793, 16.384) | 0.002 | 0.004 |
| Lateral orbitofrontal cortex | 59.701 (41.306, 78.096) | <0.001 |  | 44.138 (23.510, 64.765) | <0.001 |  | 39.117 (19.970, 58.265) | <0.001 | <0.001 |
| Medial orbitofrontal cortex | 23.968 (14.127, 33.809) | <0.001 |  | 14.469 (3.222, 25.716) | 0.012 |  | 11.743 (1.182, 22.304) | 0.029 | 0.039 |
| Paracentral lobule | 20.708 (10.318, 31.099) | <0.001 |  | 9.923 (-3.110, 22.956) | 0.135 |  | 12.796 (1.027, 24.565) | 0.033 | 0.043 |
| Parietal Lobe (mm^3^) |  |  |  |  |  |  |  |  |  |
| Postcentral gyrus | 73.782 (48.344, 99.220) | <0.001 |  | 44.791 (14.450, 75.133) | 0.004 |  | 46.344 (18.365, 74.323) | 0.001 | 0.003 |
| Superior parietal lobule | 72.583 (42.327, 102.839) | <0.001 |  | 53.249 (16.013, 90.485) | 0.005 |  | 50.509 (16.847, 84.171) | 0.003 | 0.006 |
| Inferior parietal lobule | 94.724 (56.151, 133.296) | <0.001 |  | 60.905 (15.056, 106.754) | 0.009 |  | 54.175 (12.111, 96.239) | 0.012 | 0.019 |
| Supramarginal gyrus | 79.166 (52.421, 105.910) | <0.001 |  | 67.120 (34.492, 99.747) | <0.001 |  | 63.618 (33.690, 93.546) | <0.001 | <0.001 |
| Temporal Lobe (mm^3^) |  |  |  |  |  |  |  |  |  |
| Superior temporal gyrus | 106.816 (70.699, 142.932) | <0.001 |  | 72.925 (33.255, 112.595) | <0.001 |  | 62.731 (24.989, 100.474) | 0.001 | 0.003 |
| Middle temporal gyrus | 111.460 (75.410, 147.509) | <0.001 |  | 57.142 (18.448, 95.836) | 0.004 |  | 65.706 (28.655, 102.757) | 0.001 | 0.002 |
| Inferior temporal gyrus | 95.702 (64.163, 127.241) | <0.001 |  | 82.568 (46.272, 118.864) | <0.001 |  | 78.686 (44.229, 113.143) | <0.001 | <0.001 |
| Transverse temporal gyrus | 5.779 (2.415, 9.143) | 0.001 |  | 4.732 (0.582, 8.883) | 0.026 |  | 4.374 (0.551, 8.198) | 0.025 | 0.035 |
| Fusiform gyrus | 61.604 (39.125, 84.083) | <0.001 |  | 41.799 (15.292, 68.306) | 0.002 |  | 43.471 (18.630, 68.313) | 0.001 | 0.002 |
| Entorhinal cortex | 5.749 (-0.448, 11.946) | 0.069 |  | 3.106 (-4.422, 10.634) | 0.418 |  | 4.793 (-2.238, 11.825) | 0.182 | 0.201 |
| Parahippocampal gyrus | 8.898 (2.532, 15.263) | 0.006 |  | 1.059 (-6.643, 8.760) | 0.787 |  | 2.910 (-4.188, 10.008) | 0.422 | 0.444 |
| Occipital Lobe (mm^3^) |  |  |  |  |  |  |  |  |  |
| Lateral occipital cortex | 83.746 (49.630, 117.861) | <0.001 |  | 59.533 (19.244, 99.821) | 0.004 |  | 54.864 (18.283, 91.446) | 0.003 | 0.006 |
| Cuneus | 31.937 (19.982, 43.892) | <0.001 |  | 30.303 (15.890, 44.715) | <0.001 |  | 25.621 (12.200, 39.042) | <0.001 | <0.001 |
| Precuneus | 61.370 (37.655, 85.087) | <0.001 |  | 42.434 (14.213, 70.655) | 0.003 |  | 40.554 (14.767, 66.342) | 0.002 | 0.005 |
| Lingual gyrus | 34.336 (14.504, 54.168) | 0.001 |  | 29.934 (5.736, 54.133) | 0.016 |  | 27.305 (4.986, 49.625) | 0.017 | 0.026 |
| Pericalcarine cortex | 8.704 (-1.751, 19.159) | 0.103 |  | 10.504 (-2.466, 23.473) | 0.112 |  | 4.812 (-7.136, 16.760) | 0.430 | 0.444 |
| Cingulate Cortex & Insula (mm^3^) |  |  |  |  |  |  |  |  |  |
| Rostral anterior cingulate cortex | 21.681 (12.384, 30.977) | <0.001 |  | 14.048 (3.074, 25.022) | 0.012 |  | 14.031 (3.841, 24.222) | 0.007 | 0.012 |
| Caudal anterior cingulate cortex | 18.836 (10.402, 27.270) | <0.001 |  | 13.999 (3.874, 24.125) | 0.007 |  | 12.959 (3.647, 22.270) | 0.006 | 0.012 |
| Posterior cingulate cortex | 25.251 (16.069, 34.432) | <0.001 |  | 15.613 (5.170, 26.057) | 0.004 |  | 17.043 (7.184, 26.902) | 0.001 | 0.002 |
| Isthmus of cingulate cortex | 7.774 (0.197, 15.351) | 0.044 |  | 3.078 (-6.152, 12.309) | 0.512 |  | 3.222 (-5.257, 11.700) | 0.456 | 0.456 |
| Insula | 19.620 (7.555, 31.685) | 0.002 |  | 12.250 (-2.193, 26.693) | 0.096 |  | 11.220 (-2.025, 24.465) | 0.097 | 0.120 |

^1^ Model 1 was adjusted for estimated Total Intracranial Volume.

^2^ Model 2 was further adjusted for age, sex, education, smoking status, alcohol consumption, body mass index, hypertension, diabetes, triglycerides, high-density lipoprotein cholesterol, and low-density lipoprotein cholesterol.

^3^ Model 3 was based on the covariates in Model 2, with missing data handled via multiple imputations. P-values in Model 3 were corrected for multiple comparisons using the False Discovery Rate (FDR) method; both raw P-values and FDR-corrected P-values (FDR-P) are presented.

Abbreviation: eGFR, estimated Glomerular Filtration Rate; CI, Confidence Interval; BFH-CKD study, Beijing Friendship Hospital Chronic Kidney Disease study.

**Table S8. Associations of eGFR with Cortical Thickness in META-KLS**

| Brain Measures | Model 1 ^1^ | |  | Model 2 ^2^ | |  |
| --- | --- | --- | --- | --- | --- | --- |
|  | β (95% CI) | Raw *P* value |  | β (95% CI) | Raw *P* value | FDR-*P* value |
| Frontal Lobe (μm) |  |  |  |  |  |  |
| Precentral gyrus | 22.121 (17.335, 26.906) | <0.001 |  | 7.195 (0.905, 13.484) | 0.025 | 0.303 |
| Superior frontal gyrus | 14.990 (10.997, 18.982) | <0.001 |  | 1.218 (-4.009, 6.445) | 0.648 | 0.873 |
| Rostral middle frontal gyrus | 3.136 (-0.861, 7.134) | 0.124 |  | -2.065 (-7.386, 3.257) | 0.447 | 0.765 |
| Caudal middle frontal gyrus | 12.118 (7.992, 16.244) | <0.001 |  | 4.360 (-1.180, 9.901) | 0.123 | 0.500 |
| Pars opercularis | 13.881 (9.805, 17.957) | <0.001 |  | 5.250 (-0.240, 10.739) | 0.061 | 0.377 |
| Pars triangularis | 9.489 (5.422, 13.556) | <0.001 |  | 1.138 (-4.288, 6.564) | 0.681 | 0.879 |
| Pars orbitalis | 11.388 (5.907, 16.870) | <0.001 |  | 0.578 (-6.816, 7.972) | 0.878 | 0.915 |
| Lateral orbitofrontal cortex | -2.515 (-8.809, 3.779) | 0.433 |  | -8.706 (-17.201, -0.211) | 0.045 | 0.346 |
| Medial orbitofrontal cortex | 16.628 (11.389, 21.867) | <0.001 |  | 0.722 (-6.262, 7.705) | 0.839 | 0.915 |
| Paracentral lobule | 15.751 (11.094, 20.409) | <0.001 |  | 5.147 (-1.042, 11.335) | 0.103 | 0.500 |
| Parietal Lobe (μm) |  |  |  |  |  |  |
| Postcentral gyrus | 12.435 (8.670, 16.199) | <0.001 |  | 5.588 (0.565, 10.610) | 0.029 | 0.303 |
| Superior parietal lobule | 6.544 (2.384, 10.703) | 0.002 |  | -0.122 (-5.517, 5.273) | 0.965 | 0.965 |
| Inferior parietal lobule | 6.953 (3.107, 10.799) | <0.001 |  | 0.403 (-4.740, 5.546) | 0.878 | 0.915 |
| Supramarginal gyrus | 10.162 (6.489, 13.834) | <0.001 |  | 2.322 (-2.607, 7.251) | 0.356 | 0.762 |
| Temporal Lobe (μm) |  |  |  |  |  |  |
| Superior temporal gyrus | 23.907 (19.310, 28.504) | <0.001 |  | 7.286 (1.213, 13.360) | 0.019 | 0.303 |
| Middle temporal gyrus | 10.693 (6.338, 15.048) | <0.001 |  | 3.168 (-2.718, 9.053) | 0.291 | 0.752 |
| Inferior temporal gyrus | -4.690 (-9.959, 0.578) | 0.081 |  | -3.382 (-10.568, 3.804) | 0.356 | 0.762 |
| Transverse temporal gyrus | 18.830 (12.039, 25.621) | <0.001 |  | -2.936 (-11.889, 6.017) | 0.520 | 0.769 |
| Fusiform gyrus | 6.111 (0.723, 11.500) | 0.026 |  | -2.026 (-9.348, 5.296) | 0.587 | 0.828 |
| Entorhinal cortex | 2.869 (-9.545, 15.283) | 0.650 |  | -6.601 (-23.458, 10.255) | 0.442 | 0.765 |
| Parahippocampal gyrus | 16.791 (7.495, 26.087) | <0.001 |  | 0.924 (-11.665, 13.513) | 0.886 | 0.915 |
| Occipital Lobe (μm) |  |  |  |  |  |  |
| Lateral occipital cortex | 0.587 (-3.478, 4.652) | 0.777 |  | -0.996 (-6.520, 4.529) | 0.724 | 0.897 |
| Cuneus | 5.271 (0.740, 9.801) | 0.023 |  | -2.785 (-8.861, 3.290) | 0.369 | 0.762 |
| Precuneus | 12.569 (8.513, 16.625) | <0.001 |  | 3.251 (-2.199, 8.700) | 0.242 | 0.682 |
| Lingual gyrus | 3.604 (-1.425, 8.633) | 0.160 |  | -2.665 (-9.508, 4.178) | 0.445 | 0.765 |
| Pericalcarine cortex | -4.860 (-11.851, 2.130) | 0.173 |  | -6.299 (-15.820, 3.221) | 0.195 | 0.603 |
| Cingulate Cortex & Insula (μm) |  |  |  |  |  |  |
| Rostral anterior cingulate cortex | 16.605 (11.012, 22.198) | <0.001 |  | 2.754 (-4.705, 10.213) | 0.469 | 0.765 |
| Caudal anterior cingulate cortex | 16.645 (9.983, 23.308) | <0.001 |  | 1.188 (-7.725, 10.101) | 0.794 | 0.915 |
| Posterior cingulate cortex | 12.012 (7.237, 16.786) | <0.001 |  | 2.122 (-4.365, 8.608) | 0.521 | 0.769 |
| Isthmus of cingulate cortex | -0.177 (-6.399, 6.044) | 0.955 |  | -6.283 (-14.740, 2.173) | 0.145 | 0.500 |
| Insula | 22.828 (17.348, 28.307) | <0.001 |  | 5.442 (-1.856, 12.739) | 0.144 | 0.500 |

^1^ Model 1 was unadjusted.

^2^ Model 2 was adjusted for age, sex, education, smoking status, alcohol consumption, body mass index, hypertension, diabetes, triglycerides, high-density lipoprotein cholesterol, and low-density lipoprotein cholesterol. P-values in Model 2 were corrected for multiple comparisons using the False Discovery Rate (FDR) method; both raw P-values and FDR-corrected P-values (FDR-P) are presented.

Abbreviation: eGFR, estimated Glomerular Filtration Rate; META-KLS, Multimodality MEdical imaging sTudy bAsed on Kailuan Study; CI, Confidence Interval.

**Table S9. Associations of eGFR with Cortical Thickness in UK Biobank**

| Brain Measures | Model 1 ^1^ | |  | Model 2 ^2^ | |  | Model 3 ^3^ | | |
| --- | --- | --- | --- | --- | --- | --- | --- | --- | --- |
|  | β (95% CI) | Raw *P* value |  | β (95% CI) | Raw *P* value |  | β (95% CI) | Raw *P* value | FDR-*P* value |
| Frontal Lobe (μm) |  |  |  |  |  |  |  |  |  |
| Precentral gyrus | 16.700 (12.643, 20.757) | <0.001 |  | 2.308 (-2.369, 6.986) | 0.333 |  | 2.283 (-1.982, 6.549) | 0.294 | 0.506 |
| Superior frontal gyrus | 13.819 (10.336, 17.302) | <0.001 |  | -1.155 (-5.120, 2.810) | 0.568 |  | -0.246 (-3.859, 3.367) | 0.894 | 0.955 |
| Rostral middle frontal gyrus | 8.839 (5.666, 12.012) | <0.001 |  | -2.263 (-5.909, 1.383) | 0.224 |  | -2.004 (-5.333, 1.326) | 0.238 | 0.479 |
| Caudal middle frontal gyrus | 10.615 (7.040, 14.191) | <0.001 |  | -1.151 (-5.298, 2.996) | 0.586 |  | -0.283 (-4.060, 3.495) | 0.883 | 0.955 |
| Pars opercularis | 8.389 (4.947, 11.832) | <0.001 |  | -2.108 (-6.102, 1.886) | 0.301 |  | -2.152 (-5.795, 1.491) | 0.247 | 0.479 |
| Pars triangularis | 9.126 (5.729, 12.523) | <0.001 |  | -2.450 (-6.370, 1.470) | 0.221 |  | -2.024 (-5.605, 1.558) | 0.268 | 0.489 |
| Pars orbitalis | 10.783 (6.949, 14.617) | <0.001 |  | -1.004 (-5.472, 3.463) | 0.659 |  | -0.529 (-4.613, 3.554) | 0.799 | 0.918 |
| Lateral orbitofrontal cortex | -0.536 (-3.686, 2.615) | 0.739 |  | -4.648 (-8.380, -0.915) | 0.015 |  | -4.384 (-7.790, -0.977) | 0.012 | 0.137 |
| Medial orbitofrontal cortex | 3.826 (0.095, 7.556) | 0.044 |  | -2.076 (-6.469, 2.317) | 0.354 |  | -2.518 (-6.546, 1.510) | 0.221 | 0.479 |
| Paracentral lobule | 14.331 (10.016, 18.647) | <0.001 |  | -0.625 (-5.597, 4.348) | 0.806 |  | -0.102 (-4.665, 4.461) | 0.965 | 0.997 |
| Parietal Lobe (μm) |  |  |  |  |  |  |  |  |  |
| Postcentral gyrus | 10.160 (6.829, 13.490) | <0.001 |  | 0.869 (-2.977, 4.715) | 0.658 |  | 0.939 (-2.588, 4.466) | 0.602 | 0.811 |
| Superior parietal lobule | 8.943 (5.621, 12.265) | <0.001 |  | -0.274 (-4.115, 3.566) | 0.889 |  | 0.002 (-3.509, 3.512) | 0.999 | 0.999 |
| Inferior parietal lobule | 8.471 (5.344, 11.599) | <0.001 |  | -3.678 (-7.261, -0.095) | 0.044 |  | -3.340 (-6.607, -0.074) | 0.045 | 0.280 |
| Supramarginal gyrus | 11.858 (8.442, 15.273) | <0.001 |  | -1.259 (-5.172, 2.653) | 0.528 |  | -0.556 (-4.141, 3.028) | 0.761 | 0.907 |
| Temporal Lobe (μm) |  |  |  |  |  |  |  |  |  |
| Superior temporal gyrus | 15.412 (11.724, 19.100) | <0.001 |  | 0.657 (-3.553, 4.867) | 0.760 |  | 0.624 (-3.232, 4.480) | 0.751 | 0.907 |
| Middle temporal gyrus | 7.809 (4.552, 11.065) | <0.001 |  | -1.732 (-5.523, 2.059) | 0.371 |  | -0.948 (-4.412, 2.517) | 0.592 | 0.811 |
| Inferior temporal gyrus | 4.302 (1.270, 7.333) | 0.005 |  | -2.827 (-6.395, 0.740) | 0.120 |  | -2.149 (-5.404, 1.105) | 0.196 | 0.479 |
| Transverse temporal gyrus | -2.733 (-8.032, 2.567) | 0.312 |  | -5.135 (-11.381, 1.111) | 0.107 |  | -4.693 (-10.439, 1.052) | 0.109 | 0.377 |
| Fusiform gyrus | 7.246 (4.191, 10.300) | <0.001 |  | -1.778 (-5.340, 1.784) | 0.328 |  | -1.490 (-4.739, 1.760) | 0.369 | 0.602 |
| Entorhinal cortex | 18.404 (12.092, 24.716) | <0.001 |  | 1.369 (-6.038, 8.776) | 0.717 |  | 4.014 (-2.775, 10.803) | 0.247 | 0.479 |
| Parahippocampal gyrus | 9.494 (3.705, 15.283) | 0.001 |  | -0.921 (-7.642, 5.799) | 0.788 |  | -1.100 (-7.291, 5.091) | 0.728 | 0.907 |
| Occipital Lobe (μm) |  |  |  |  |  |  |  |  |  |
| Lateral occipital cortex | 1.594 (-1.225, 4.412) | 0.268 |  | -2.442 (-5.745, 0.861) | 0.147 |  | -2.030 (-5.049, 0.989) | 0.187 | 0.479 |
| Cuneus | -0.280 (-3.242, 2.682) | 0.853 |  | -2.778 (-6.254, 0.697) | 0.117 |  | -2.118 (-5.310, 1.073) | 0.193 | 0.479 |
| Precuneus | 9.819 (6.471, 13.167) | <0.001 |  | -2.225 (-6.101, 1.650) | 0.260 |  | -1.460 (-4.996, 2.076) | 0.418 | 0.649 |
| Lingual gyrus | -1.020 (-4.072, 2.032) | 0.512 |  | -4.175 (-7.772, -0.578) | 0.023 |  | -4.154 (-7.442, -0.866) | 0.013 | 0.137 |
| Pericalcarine cortex | -5.330 (-8.436, -2.223) | 0.001 |  | -3.319 (-6.950, 0.312) | 0.073 |  | -2.886 (-6.234, 0.462) | 0.091 | 0.353 |
| Cingulate Cortex & Insula (μm) |  |  |  |  |  |  |  |  |  |
| Rostral anterior cingulate cortex | -0.027 (-3.924, 3.869) | 0.989 |  | -7.756 (-12.322, -3.189) | 0.001 |  | -6.292 (-10.497, -2.086) | 0.003 | 0.105 |
| Caudal anterior cingulate cortex | 3.772 (-1.956, 9.501) | 0.197 |  | -5.731 (-12.428, 0.966) | 0.094 |  | -5.741 (-11.901, 0.419) | 0.068 | 0.350 |
| Posterior cingulate cortex | 4.986 (1.396, 8.576) | 0.007 |  | -5.042 (-9.257, -0.826) | 0.019 |  | -3.393 (-7.240, 0.453) | 0.084 | 0.353 |
| Isthmus of cingulate cortex | 4.309 (0.566, 8.052) | 0.024 |  | -5.947 (-10.295, -1.599) | 0.007 |  | -4.394 (-8.396, -0.393) | 0.031 | 0.243 |
| Insula | 7.229 (3.689, 10.770) | <0.001 |  | -1.534 (-5.660, 2.592) | 0.466 |  | -1.172 (-4.946, 2.603) | 0.543 | 0.801 |

^1^ Model 1 was unadjusted.

^2^ Model 2 was adjusted for age, sex, education, smoking status, alcohol consumption, body mass index, hypertension, diabetes, triglycerides, high-density lipoprotein cholesterol, and low-density lipoprotein cholesterol.

^3^ Model 3 was based on the covariates in Model 2, with missing data handled via multiple imputations. P-values in Model 3 were corrected for multiple comparisons using the False Discovery Rate (FDR) method; both raw P-values and FDR-corrected P-values (FDR-P) are presented.

Abbreviation: eGFR, estimated Glomerular Filtration Rate; CI, Confidence Interval.

**Table S10. Associations of eGFR with Cortical Thickness in BFH-CKD**

| Brain Measures | Model 1 ^1^ | |  | Model 2 ^2^ | |  | Model 3 ^3^ | | |
| --- | --- | --- | --- | --- | --- | --- | --- | --- | --- |
|  | β (95% CI) | Raw *P* value |  | β (95% CI) | Raw *P* value |  | β (95% CI) | Raw *P* value | FDR-*P* value |
| Frontal Lobe (μm) |  |  |  |  |  |  |  |  |  |
| Precentral gyrus | 5.520 (2.411, 8.628) | 0.001 |  | 2.175 (-1.513, 5.862) | 0.247 |  | 2.032 (-1.322, 5.385) | 0.235 | 0.270 |
| Superior frontal gyrus | 7.125 (4.519, 9.731) | <0.001 |  | 3.632 (0.500, 6.765) | 0.023 |  | 3.818 (0.966, 6.670) | 0.009 | 0.021 |
| Rostral middle frontal gyrus | 2.020 (-0.404, 4.443) | 0.102 |  | -1.171 (-4.083, 1.742) | 0.430 |  | -0.195 (-2.870, 2.480) | 0.887 | 0.887 |
| Caudal middle frontal gyrus | 5.162 (2.308, 8.015) | <0.001 |  | 2.255 (-1.246, 5.757) | 0.206 |  | 1.967 (-1.215, 5.149) | 0.227 | 0.269 |
| Pars opercularis | 6.384 (3.836, 8.932) | <0.001 |  | 4.030 (1.073, 6.986) | 0.008 |  | 3.425 (0.659, 6.191) | 0.015 | 0.031 |
| Pars triangularis | 6.540 (4.014, 9.066) | <0.001 |  | 3.186 (0.242, 6.130) | 0.034 |  | 3.736 (1.000, 6.472) | 0.007 | 0.019 |
| Pars orbitalis | 9.272 (6.078, 12.466) | <0.001 |  | 4.884 (1.156, 8.612) | 0.010 |  | 5.336 (1.923, 8.748) | 0.002 | 0.009 |
| Lateral orbitofrontal cortex | 7.004 (4.355, 9.652) | <0.001 |  | 5.096 (1.943, 8.249) | 0.002 |  | 4.523 (1.645, 7.400) | 0.002 | 0.009 |
| Medial orbitofrontal cortex | 6.987 (4.228, 9.747) | <0.001 |  | 4.520 (1.320, 7.720) | 0.006 |  | 4.448 (1.430, 7.467) | 0.004 | 0.012 |
| Paracentral lobule | 5.174 (2.377, 7.971) | <0.001 |  | 3.617 (0.208, 7.026) | 0.038 |  | 3.192 (0.050, 6.334) | 0.047 | 0.072 |
| Parietal Lobe (μm) |  |  |  |  |  |  |  |  |  |
| Postcentral gyrus | 5.846 (3.275, 8.417) | <0.001 |  | 3.829 (0.711, 6.947) | 0.016 |  | 3.359 (0.506, 6.211) | 0.021 | 0.038 |
| Superior parietal lobule | 4.672 (2.247, 7.097) | <0.001 |  | 2.988 (0.095, 5.880) | 0.043 |  | 2.793 (0.103, 5.483) | 0.042 | 0.068 |
| Inferior parietal lobule | 6.246 (3.770, 8.722) | <0.001 |  | 4.513 (1.488, 7.538) | 0.004 |  | 4.529 (1.727, 7.332) | 0.002 | 0.008 |
| Supramarginal gyrus | 8.528 (6.164, 10.892) | <0.001 |  | 6.063 (3.262, 8.865) | <0.001 |  | 6.167 (3.546, 8.787) | <0.001 | <0.001 |
| Temporal Lobe (μm) |  |  |  |  |  |  |  |  |  |
| Superior temporal gyrus | 12.279 (9.342, 15.216) | <0.001 |  | 8.682 (5.335, 12.029) | <0.001 |  | 8.570 (5.495, 11.646) | <0.001 | <0.001 |
| Middle temporal gyrus | 8.500 (5.711, 11.289) | <0.001 |  | 5.077 (1.744, 8.410) | 0.003 |  | 5.591 (2.508, 8.674) | <0.001 | 0.003 |
| Inferior temporal gyrus | 7.961 (4.854, 11.068) | <0.001 |  | 5.518 (1.794, 9.241) | 0.004 |  | 5.267 (1.847, 8.688) | 0.003 | 0.009 |
| Transverse temporal gyrus | 7.541 (4.155, 10.926) | <0.001 |  | 6.328 (2.161, 10.494) | 0.003 |  | 6.518 (2.675, 10.360) | 0.001 | 0.006 |
| Fusiform gyrus | 6.675 (4.007, 9.342) | <0.001 |  | 3.298 (0.075, 6.521) | 0.045 |  | 3.681 (0.692, 6.670) | 0.016 | 0.031 |
| Entorhinal cortex | 7.833 (2.253, 13.412) | 0.006 |  | 7.853 (1.054, 14.652) | 0.024 |  | 8.075 (1.821, 14.330) | 0.011 | 0.025 |
| Parahippocampal gyrus | 8.098 (3.312, 12.884) | 0.001 |  | 2.129 (-3.586, 7.844) | 0.464 |  | 3.336 (-2.024, 8.696) | 0.223 | 0.269 |
| Occipital Lobe (μm) |  |  |  |  |  |  |  |  |  |
| Lateral occipital cortex | 3.858 (1.163, 6.552) | 0.005 |  | 1.684 (-1.588, 4.955) | 0.312 |  | 2.230 (-0.831, 5.291) | 0.153 | 0.198 |
| Cuneus | 2.232 (-0.100, 4.564) | 0.061 |  | 1.733 (-1.110, 4.576) | 0.231 |  | 1.563 (-1.089, 4.215) | 0.248 | 0.275 |
| Precuneus | 5.005 (2.394, 7.617) | <0.001 |  | 1.915 (-1.133, 4.963) | 0.217 |  | 2.133 (-0.709, 4.975) | 0.141 | 0.190 |
| Lingual gyrus | 1.236 (-1.560, 4.032) | 0.385 |  | 1.501 (-2.070, 5.073) | 0.409 |  | 1.363 (-1.850, 4.575) | 0.406 | 0.434 |
| Pericalcarine cortex | -4.084 (-7.345, -0.822) | 0.014 |  | -2.976 (-6.984, 1.032) | 0.145 |  | -3.724 (-7.440, -0.008) | 0.050 | 0.073 |
| Cingulate Cortex & Insula (μm) |  |  |  |  |  |  |  |  |  |
| Rostral anterior cingulate cortex | 6.727 (2.969, 10.484) | 0.001 |  | 3.849 (-0.684, 8.382) | 0.096 |  | 3.712 (-0.472, 7.896) | 0.082 | 0.116 |
| Caudal anterior cingulate cortex | 9.892 (5.974, 13.810) | <0.001 |  | 5.439 (0.687, 10.191) | 0.025 |  | 6.151 (1.799, 10.503) | 0.006 | 0.016 |
| Posterior cingulate cortex | 6.915 (3.955, 9.876) | <0.001 |  | 2.944 (-0.500, 6.389) | 0.094 |  | 3.646 (0.448, 6.844) | 0.025 | 0.044 |
| Isthmus of cingulate cortex | 4.051 (0.713, 7.388) | 0.018 |  | 1.365 (-2.654, 5.384) | 0.505 |  | 1.365 (-2.375, 5.105) | 0.474 | 0.490 |
| Insula | 13.670 (9.843, 17.497) | <0.001 |  | 8.797 (4.582, 13.013) | <0.001 |  | 8.336 (4.382, 12.291) | <0.001 | <0.001 |

^1^ Model 1 was unadjusted.

^2^ Model 2 was adjusted for age, sex, education, smoking status, alcohol consumption, body mass index, hypertension, diabetes, triglycerides, high-density lipoprotein cholesterol, and low-density lipoprotein cholesterol.

^3^ Model 3 was based on the covariates in Model 2, with missing data handled via multiple imputations. P-values in Model 3 were corrected for multiple comparisons using the False Discovery Rate (FDR) method; both raw P-values and FDR-corrected P-values (FDR-P) are presented.

Abbreviation: eGFR, estimated Glomerular Filtration Rate; CI, Confidence Interval; BFH-CKD study, Beijing Friendship Hospital Chronic Kidney Disease study.

**Table S11. Associations of eGFR Variability (VIM) with Whole-Brain and Subcortical Volumes in META-KLS**

| Brain Measures | Model 1 ^1^ | |  | Model 2 ^2^ | |  |
| --- | --- | --- | --- | --- | --- | --- |
|  | β (95% CI) | Raw *P* value |  | β (95% CI) | Raw *P* value | FDR-*P* value |
| Whole-brain metrics (10^3^ mm^3^) |  |  |  |  |  |  |
| Grey+white matter | -9.761 (-13.701, -5.821) | <0.001 |  | -3.935 (-8.176, 0.306) | 0.069 | 0.159 |
| Grey matter | -3.203 (-5.329, -1.077) | 0.003 |  | -1.850 (-4.161, 0.461) | 0.117 | 0.159 |
| White matter | -6.432 (-9.077, -3.787) | <0.001 |  | -1.911 (-4.571, 0.748) | 0.159 | 0.159 |
| Subcortical structures (mm^3^) |  |  |  |  |  |  |
| Caudate | -4.398 (-79.601 70.804) | 0.909 |  | -42.705 (-129.137, 43.727) | 0.333 | 0.977 |
| Putamen | -11.637 (-121.189, 97.915) | 0.835 |  | -28.159 (-154.110, 97.792) | 0.661 | 0.977 |
| Pallidum | 19.862 (-21.113, 60.837) | 0.342 |  | -3.136 (-49.534, 43.262) | 0.895 | 0.977 |
| Hippocampus | 9.608 (-67.588, 86.803) | 0.807 |  | -30.508 (-119.265, 58.249) | 0.500 | 0.977 |
| Amygdala | -2.515 (-35.197, 30.166) | 0.880 |  | -11.355 (-49.216, 26.505) | 0.556 | 0.977 |
| Thalamus | 9.604 (-174.247, 193.455) | 0.918 |  | -7.262 (-220.783, 206.259) | 0.947 | 0.977 |
| Accumbens | -2.937 (-13.026, 7.152) | 0.568 |  | 0.172 (-11.345, 11.690) | 0.977 | 0.977 |

^1^ Model 1 was adjusted for estimated Total Intracranial Volume.

^2^ Model 2 was further adjusted for age, sex, education, smoking status, alcohol consumption, body mass index, hypertension, diabetes, triglycerides, high-density lipoprotein cholesterol, low-density lipoprotein cholesterol, and baseline eGFR. P-values in Model 2 were corrected for multiple comparisons using the False Discovery Rate (FDR) method; both raw P-values and FDR-corrected P-values (FDR-P) are presented.

Abbreviation: eGFR, estimated Glomerular Filtration Rate; META-KLS, Multimodality MEdical imaging sTudy bAsed on Kailuan Study; CI, Confidence Interval.

**Table S12. Associations of eGFR Variability (VIM) and Cortical Volumes in META-KLS**

| Brain Measures | Model 1 ^1^ | |  | Model 2 ^2^ | |  |
| --- | --- | --- | --- | --- | --- | --- |
|  | β (95% CI) | Raw *P* value |  | β (95% CI) | Raw *P* value | FDR-*P* value |
| Frontal Lobe (mm^3^) |  |  |  |  |  |  |
| Precentral gyrus | -55.882 (-118.876, 7.112) | 0.082 |  | -26.980 (-98.104, 44.144) | 0.457 | 0.963 |
| Superior frontal gyrus | -155.744 (-256.999, -54.489) | 0.003 |  | -26.630 (-138.927, 85.667) | 0.642 | 0.963 |
| Rostral middle frontal gyrus | -66.633 (-134.313, 1.047) | 0.054 |  | -6.893 (-84.128, 70.342) | 0.861 | 0.963 |
| Caudal middle frontal gyrus | -78.280 (-124.333, -32.228) | 0.001 |  | -52.011 (-104.641, 0.620) | 0.053 | 0.546 |
| Pars opercularis | -24.450 (-55.604, 6.704) | 0.124 |  | 6.808 (-28.577, 42.192) | 0.706 | 0.963 |
| Pars triangularis | -12.832 (-45.028, 19.363) | 0.434 |  | -1.401 (-38.322, 35.520) | 0.941 | 0.963 |
| Pars orbitalis | -1.017 (-14.809, 12.774) | 0.885 |  | 12.881 (-2.941, 28.703) | 0.111 | 0.571 |
| Lateral orbitofrontal cortex | -28.650 (-66.623, 9.323) | 0.139 |  | -14.144 (-58.248, 29.960) | 0.529 | 0.963 |
| Medial orbitofrontal cortex | -24.559 (-43.849, -5.269) | 0.013 |  | 1.612 (-20.183, 23.407) | 0.885 | 0.963 |
| Paracentral lobule | -23.387 (-45.698, -1.075) | 0.040 |  | -4.294 (-29.690, 21.101) | 0.740 | 0.963 |
| Parietal Lobe (mm^3^) |  |  |  |  |  |  |
| Postcentral gyrus | -24.529 (-77.678, 28.621) | 0.365 |  | -10.864 (-72.034, 50.306) | 0.728 | 0.963 |
| Superior parietal lobule | -79.411 (-143.438, -15.385) | 0.015 |  | -60.442 (-133.358, 12.474) | 0.104 | 0.571 |
| Inferior parietal lobule | -144.054 (-216.697, -71.411) | <0.001 |  | -84.372 (-165.884, -2.860) | 0.043 | 0.546 |
| Supramarginal gyrus | -42.629 (-101.225, 15.968) | 0.154 |  | -13.542 (-80.760, 53.675) | 0.693 | 0.963 |
| Temporal Lobe (mm^3^) |  |  |  |  |  |  |
| Superior temporal gyrus | -80.898 (-152.606, -9.190) | 0.027 |  | -16.523 (-97.943, 64.897) | 0.691 | 0.963 |
| Middle temporal gyrus | -174.830 (-246.065, -103.595) | <0.001 |  | -89.951 (-169.071, -10.831) | 0.026 | 0.546 |
| Inferior temporal gyrus | -39.560 (-109.936, 30.816) | 0.270 |  | -4.501 (-85.931, 76.928) | 0.914 | 0.963 |
| Transverse temporal gyrus | -2.808 (-10.748, 5.132) | 0.488 |  | 2.253 (-6.783, 11.288) | 0.625 | 0.963 |
| Fusiform gyrus | -48.237 (-92.490, -3.983) | 0.033 |  | -9.612 (-59.668, 40.444) | 0.706 | 0.963 |
| Entorhinal cortex | -3.436 (-19.028, 12.155) | 0.666 |  | 7.307 (-10.638, 25.253) | 0.425 | 0.963 |
| Parahippocampal gyrus | -8.972 (-23.078, 5.1333) | 0.212 |  | 3.186 (-13.043, 19.415) | 0.700 | 0.963 |
| Occipital Lobe (mm^3^) |  |  |  |  |  |  |
| Lateral occipital cortex | -4.931 (-70.837, 60.975) | 0.883 |  | 38.277 (-37.749, 114.303) | 0.323 | 0.911 |
| Cuneus | -5.168 (-32.461, 22.124) | 0.710 |  | 0.744 (-30.818, 32.307) | 0.963 | 0.963 |
| Precuneus | -59.867 (-107.939, -11.796) | 0.015 |  | -28.768 (-83.582 26.045) | 0.303 | 0.911 |
| Lingual gyrus | 11.754 (-36.121, 59.629) | 0.630 |  | 28.447 (-26.574, 83.468) | 0.311 | 0.911 |
| Pericalcarine cortex | 9.906 (-16.663, 36.476) | 0.465 |  | -2.959 (-33.766, 27.849) | 0.851 | 0.963 |
| Cingulate Cortex & Insula (mm^3^) |  |  |  |  |  |  |
| Rostral anterior cingulate cortex | -23.412 (-43.999, -2.825) | 0.026 |  | -13.353 (-37.158, 10.451) | 0.271 | 0.911 |
| Caudal anterior cingulate cortex | -28.162 (-47.883, -8.441) | 0.005 |  | -20.145 (-42.667, 2.376) | 0.080 | 0.571 |
| Posterior cingulate cortex | -35.540 (-61.406, -9.673) | 0.007 |  | -16.274 (-45.252, 12.704) | 0.271 | 0.911 |
| Isthmus of cingulate cortex | -5.969 (-22.393, 10.455) | 0.476 |  | 0.543 (-18.209, 19.296) | 0.955 | 0.963 |
| Insula | -8.218 (-34.187, 17.752) | 0.535 |  | 1.224 (-28.359, 30.807) | 0.935 | 0.963 |

^1^ Model 1 was adjusted for estimated Total Intracranial Volume.

^2^ Model 2 was further adjusted for age, sex, education, smoking status, alcohol consumption, body mass index, hypertension, diabetes, triglycerides, high-density lipoprotein cholesterol, low-density lipoprotein cholesterol, and baseline eGFR. P-values in Model 2 were corrected for multiple comparisons using the False Discovery Rate (FDR) method; both raw P-values and FDR-corrected P-values (FDR-P) are presented.

Abbreviation: eGFR, estimated Glomerular Filtration Rate; META-KLS, Multimodality MEdical imaging sTudy bAsed on Kailuan Study; CI, Confidence Interval.

**Table S13. Associations of eGFR Variability (VIM) and Cortical Thickness in META-KLS**

| Brain Measures | Model 1 ^1^ | |  | Model 2 ^2^ | |  |
| --- | --- | --- | --- | --- | --- | --- |
|  | β (95% CI) | Raw P value |  | β (95% CI) | Raw *P* value | FDR-*P* value |
| Frontal Lobe (μm) |  |  |  |  |  |  |
| Precentral gyrus | -10.657 (-17.191, -4.124) | 0.001 |  | -0.633 (-7.763, 6.497) | 0.862 | 0.898 |
| Superior frontal gyrus | -3.303 (-8.728, 2.122) | 0.233 |  | 1.662 (-4.263, 7.586) | 0.582 | 0.898 |
| Rostral middle frontal gyrus | 4.000 (-1.298, 9.298) | 0.139 |  | 3.979 (-2.052, 10.009) | 0.196 | 0.898 |
| Caudal middle frontal gyrus | -6.203 (-11.755, -0.652) | 0.029 |  | -1.539 (-7.835, 4.756) | 0.631 | 0.898 |
| Pars opercularis | -5.004 (-10.534, 0.526) | 0.076 |  | 2.293 (-3.945, 8.531) | 0.471 | 0.898 |
| Pars triangularis | -2.232 (-7.690, 3.227) | 0.423 |  | -0.519 (-6.684, 5.647) | 0.869 | 0.898 |
| Pars orbitalis | -2.462 (-9.798, 4.874) | 0.510 |  | 1.275 (-7.118, 9.668) | 0.766 | 0.898 |
| Lateral orbitofrontal cortex | 8.184 (-0.153, 16.521) | 0.054 |  | 2.848 (-6.777, 12.473) | 0.562 | 0.898 |
| Medial orbitofrontal cortex | -2.616 (-9.681, 4.450) | 0.468 |  | 4.600 (-3.313, 12.513) | 0.254 | 0.898 |
| Paracentral lobule | -7.805 (-14.076 -1.534) | 0.015 |  | -1.235 (-008.254, 5.785) | 0.730 | 0.898 |
| Parietal Lobe (μm) |  |  |  |  |  |  |
| Postcentral gyrus | -7.480 (-12.548, -2.412) | 0.004 |  | -2.482 (-8.181, 3.218) | 0.393 | 0.898 |
| Superior parietal lobule | -5.011 (-10.546, 0.525) | 0.076 |  | -1.260 (-7.386, 4.867) | 0.687 | 0.898 |
| Inferior parietal lobule | -4.295 (-9.421, 0.832) | 0.101 |  | -1.473 (-7.310, 4.364) | 0.621 | 0.898 |
| Supramarginal gyrus | -5.355 (-10.280, -0.430) | 0.033 |  | -2.390 (-7.988, 3.209) | 0.403 | 0.898 |
| Temporal Lobe (μm) |  |  |  |  |  |  |
| Superior temporal gyrus | -12.585 (-18.927, -6.242) | <0.001 |  | -4.565 (-11.459, 2.330) | 0.194 | 0.898 |
| Middle temporal gyrus | -6.667 (-12.499, -0.835) | 0.025 |  | -3.318 (-10.002, 3.366) | 0.330 | 0.898 |
| Inferior temporal gyrus | 3.648 (-3.359, 10.656) | 0.307 |  | -1.206 (-9.376, 6.964) | 0.772 | 0.898 |
| Transverse temporal gyrus | -2.353 (-11.496, 6.789) | 0.614 |  | 1.983 (-8.181, 12.146) | 0.702 | 0.898 |
| Fusiform gyrus | -1.837 (-9.002, 5.329) | 0.615 |  | -1.615 (-9.911, 6.681) | 0.703 | 0.898 |
| Entorhinal cortex | 0.213 (-16.292, 16.718) | 0.980 |  | 2.226 (-16.969, 21.421) | 0.820 | 0.898 |
| Parahippocampal gyrus | -3.731 (-16.147, 8.685) | 0.556 |  | 1.198 (-13.096, 15.492) | 0.869 | 0.898 |
| Occipital Lobe (μm) |  |  |  |  |  |  |
| Lateral occipital cortex | 0.481 (-4.911, 5.873) | 0.861 |  | 4.007 (-2.253, 10.267) | 0.209 | 0.898 |
| Cuneus | -2.241 (-8.245, 3.763) | 0.464 |  | 0.087 (-6.803, 6.977) | 0.980 | 0.980 |
| Precuneus | -7.149 (-12.625, -1.673) | 0.011 |  | -2.141 (-8.339, 4.057) | 0.498 | 0.898 |
| Lingual gyrus | -1.457 (-8.136, 5.222) | 0.669 |  | -1.738 (-9.500, 6.025) | 0.661 | 0.898 |
| Pericalcarine cortex | -0.859 (-10.151, 8.433) | 0.856 |  | -4.690 (-15.495, 6.115) | 0.395 | 0.898 |
| Cingulate Cortex & Insula (μm) |  |  |  |  |  |  |
| Rostral anterior cingulate cortex | -9.489 (-16.965, -2.012) | 0.013 |  | -9.035 (-17.470, -0.600) | 0.036 | 0.898 |
| Caudal anterior cingulate cortex | -2.395 (-11.366, 6.576) | 0.601 |  | 3.949 (-6.229, 14.127) | 0.447 | 0.898 |
| Posterior cingulate cortex | -1.691 (-8.101, 4.720) | 0.605 |  | 1.574 (-5.771, 8.919) | 0.674 | 0.898 |
| Isthmus of cingulate cortex | 7.573 (-0.676, 15.822) | 0.072 |  | 6.670 (-2.933, 16.273) | 0.173 | 0.898 |
| Insula | -12.203 (-19.659, -4.746) | 0.001 |  | -4.184 (-12.454, 4.086) | 0.321 | 0.898 |

^1^ Model 1 was unadjusted.

^2^ Model 2 was adjusted for age, sex, education, smoking status, alcohol consumption, body mass index, hypertension, diabetes, triglycerides, high-density lipoprotein cholesterol, low-density lipoprotein cholesterol, and baseline eGFR. P-values in Model 2 were corrected for multiple comparisons using the False Discovery Rate (FDR) method; both raw P-values and FDR-corrected P-values (FDR-P) are presented.

Abbreviation: eGFR, estimated Glomerular Filtration Rate; META-KLS, Multimodality MEdical imaging sTudy bAsed on Kailuan Study; CI, Confidence Interval.

**Figure S1. Flow Diagram of Participant Selection**

Panel A shows the participant selection process for META-KLS, panel B for UK Biobank, and panel C for BFH-CKD.

Abbreviation: META-KLS, Multimodality MEdical imaging sTudy bAsed on Kailuan Study; MRI, Magnetic Resonance Imaging; eTIV, estimated Total Intracranial Volume; SD, Standard Deviation; BFH-CKD study, Beijing Friendship Hospital Chronic Kidney Disease study.

**Figure S2. Associations of eGFR with Cortical Thickness across the META-KLS, UK Biobank, and BFH-CKD Cohorts**

Forest plot summarized cortical thickness with nominally (raw P<0.05) or FDR-significant association with eGFR in each cohort. β coefficients and 95% CIs were calculated after adjustment for age, sex, education, smoking status, alcohol consumption, body mass index, hypertension, diabetes, triglycerides, high-density lipoprotein cholesterol, and low-density lipoprotein cholesterol. Complete results for all cortical thickness measures are provided in Supplementary Table S8, Table S9, and Table S10.

Abbreviation: META-KLS, Multimodality MEdical imaging sTudy bAsed on Kailuan Study; BFH-CKD study, Beijing Friendship Hospital Chronic Kidney Disease study; FDR, False Discovery Rate; CI, confidence interval.

**Figure S3. Mediation Analysis of Brain Structures in the Association Between Cumulative eGFR and Cognitive Function in META-KLS**

All models were adjusted for age, sex, education, smoking status, alcohol consumption, body mass index, hypertension, diabetes, triglycerides, high-density lipoprotein cholesterol, low-density lipoprotein cholesterol, and estimated total intracranial volume.

Abbreviation: META-KLS, Multimodality MEdical imaging sTudy bAsed on Kailuan Study; eGFR, estimated Glomerular Filtration Rate; MoCA, Montreal Cognitive Assessment; DE,direct effect; IE, indirect effect.

**Figure S4. Mediation Analysis of Brain Structures in the Association Between eGFR Variability and Cognitive Function in META-KLS**

All models were adjusted for age, sex, education, smoking status, alcohol consumption, body mass index, hypertension, diabetes, triglycerides, high-density lipoprotein cholesterol, low-density lipoprotein cholesterol and baseline eGFR. Models for cortical volume were additionally adjusted for estimated total intracranial volume.

Abbreviation: META-KLS, Multimodality MEdical imaging sTudy bAsed on Kailuan Study; eGFR, estimated Glomerular Filtration Rate; MoCA, Montreal Cognitive Assessment; DE,direct effect; IE, indirect effect.
